# Advances in antimicrobial peptides: promising cancer treatments and vaccines

**DOI:** 10.3389/fmed.2026.1783547

**Published:** 2026-03-31

**Authors:** Gehane Ghaly, Hatem Tallima, Tamer Shoeib

**Affiliations:** Department of Chemistry, The American University in Cairo, New Cairo, Egypt

**Keywords:** anticancer drugs, anticancer peptides, antimicrobial peptides, cancer immunotherapy, immunogenic cell death, immunomodulators

## Abstract

Antimicrobial peptides (AMPs), long recognized for their broad-spectrum antimicrobial activity, have recently gained prominence as versatile anticancer agents. This review synthesizes recent advances positioning anticancer peptides (ACPs) at the forefront of novel oncological strategies, driven by their unique biophysical properties. Their cationic, amphipathic architecture enables selective electrostatic interactions with negatively charged malignant cell membranes, resulting in targeted, rapid membrane disruption and cell lysis. Beyond direct membrane effects, ACPs exert multifaceted intracellular actions including inhibition of DNA replication and protein synthesis, induction of mitochondrial dysfunction, and suppression of tumor angiogenesis. Increasingly, AMPs are recognized as potent immunomodulators capable of remodeling the tumor microenvironment. They induce immunogenic cell death, functioning as *in situ* vaccines that prime systemic antitumor immunity through intrinsic adjuvant effects that enhance antigen presentation. This review highlights clinically relevant AMPs categorized by Food and Drug Administration and European Medicines Agency approval status, illustrating the diversity of their therapeutic targets and mechanisms. We also critically examine key challenges hindering clinical translation, such as proteolytic instability, hemolytic toxicity, and suboptimal pharmacokinetics, and evaluate emerging solutions, including peptide engineering, nanoparticle-based delivery, and advanced conjugation strategies to improve stability, tumor specificity, and accumulation. Crucially, the integration of AMPs into combination regimens with conventional and immunotherapeutic agents presents a transformative strategy to overcome drug resistance and immune evasion. Nevertheless, clinical evidence remains limited, with most studies confined to early-phase trials. Ongoing efforts in optimizing peptide stability, developing targeted delivery systems, and identifying predictive biomarkers are essential to translate the promising preclinical profile of AMPs into clinically viable cancer therapeutics. With sustained interdisciplinary innovation and rigorous validation, AMPs are poised to become integral components of next-generation precision oncology.

## 1 Introduction to cancer therapy

Cancer remains a leading cause of mortality worldwide, despite significant progress in early detection and treatment. Conventional therapeutic modalities including surgery, cytotoxic chemotherapy, radiation, and targeted therapy, are constrained by limited specificity, systemic toxicity, and the emergence of drug resistance. The advent of cancer immunotherapy (CI), particularly immune checkpoint inhibitors, has marked a paradigm shift in oncology. However, challenges such as variable response rates and immune-related adverse events underscore the need for complementary strategies. Within this landscape, a distinct class of antimicrobial peptides (AMPs), exhibiting anticancer activity are recognized as anticancer peptides (ACPs) has garnered increasing attention as a promising alternative or adjunct to existing regimens (1, 2).

These naturally derived or synthetic peptides offer distinct mechanistic advantages, including selective cytotoxicity toward malignant cells, rapid membrane-disruptive action, and the capacity to modulate antitumor immunity. Nonetheless, the literature lacks an integrated synthesis that captures both the recent mechanistic insights into ACPs and the parallel advances in peptide engineering aimed at overcoming longstanding translational barriers. This review addresses that gap by providing a comprehensive overview of the evolving role of AMPs in oncology with emphasis on their multifunctional mechanisms, immunomodulatory properties, and clinical development status. Additionally, we examine emerging strategies to enhance peptide stability, tumor specificity, and pharmacokinetic profiles, thereby framing the trajectory of AMPs as a clinically viable class of cancer therapeutics.

## 2 AMPs: characteristics and modes of action of ACPs on cancer cells

AMPs are increasingly recognized for their highly efficient, selective targeting and elimination of cancer cells, with no adverse cytotoxic effects on normal cells, and with no developed drug resistance (1). AMPs selectivity is essentially due to their cationic nature, targeting cells exposing negatively charged phosphatidyl serine, a hallmark of cancer cells, as mentioned in section 2.2. All modes of binding follow electrostatic interactions, not available with healthy normal cells, which display slightly alkaline pH of 7.0–7.2. Conversely, the average pH at the plasma membrane of human cancer cells is typically acidic, ranging between 6.7 and 7.1. In highly aggressive or poorly perfused regions of a tumor, this pH can drop to as low as 6.0–6.5. AMPs have been reported to be increasingly recognized for their highly efficient, selective targeting and elimination of cancer cells, with no adverse cytotoxic effects on normal cells, and with no developed drug resistance (1). However, high concentrations of peptides can still result in cytotoxicity to normal cells. This issue has been addressed through different approaches, including addition of a small targeting sequence to the peptide, which interacts with the cancer cell expressed surface molecules, or modifying the peptide amino acid sequence through substitution of amino acids. The first approach, besides being costly, depends on maintained expression of the targeted surface molecules by the cancer cell, while the second approach may alter the characteristics of the peptide related to its anticancer efficacy, such as charge and hydrophobicity (3–5).

### 2.1 Characteristics of AMPs

Natural AMPs are cationic small peptides, that form an important part of organisms’ innate immunity, and participate in regulating the immune system against bacterial, viral or fungal infections (2, 6, 7). They are formed from 10 to 100 amino acid residues, with high contribution of hydrophobic ones. The most common conformation for natural ACPs is α-helix, while some still acquire β-sheet conformation. Synthetic ACPs, on the other hand, can be a hybrid of both conformations. As the outer membrane leaflet in cancer cells shows higher expression of phosphatidylserine and O-glycosylated mucins, they possess highly negatively charged membranes compared to normal cells, making them an inevitable target to electrostatic attraction and hence attack by the cationic ACPs, resulting in severe cancer cell damage and death, through various mechanisms, without being hydrolyzed themselves by serum peptidases (8–11).

### 2.2 Modes of action of ACPs

AMPs can destroy cancer cells through various mechanisms. Their electrostatic binding to the negatively charged cell membrane of cancer cells creates transmembrane channels, and demolishes the membrane integrity. They can also interfere with intracellular processes, such as DNA replication, protein synthesis and folding, induce damage to cancer cells cytoskeleton, inhibit tumor angiogenesis, or attack the mitochondria resulting in cancer cells apoptosis and necrosis (11).

**Figure 1:**
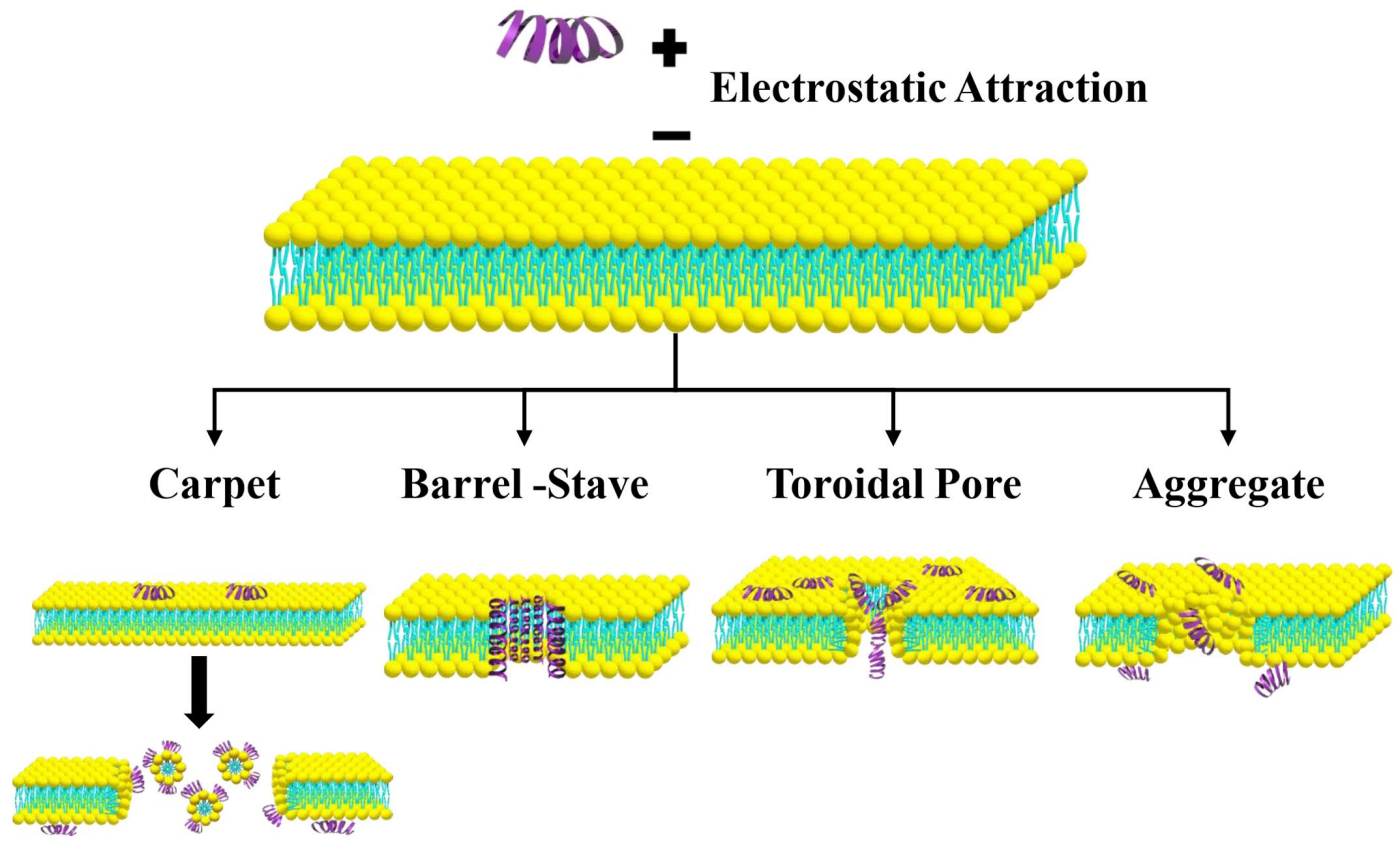
ACPs modes of action.

ACPs interaction mechanisms with cancer cell membranes have been elucidated to occur through four main models: carpet, barrel-stave, toroidal pore and aggregate models (Table 1; Figure 1). The required active concentration for the same type of peptide may vary among the various models (11). It is important to note that these mechanisms are not mutually exclusive; a single peptide may employ multiple mechanisms simultaneously or sequentially depending on factors such as peptide concentration, membrane lipid composition, and the tumor microenvironment (TME). Understanding this mechanistic plasticity has important therapeutic implications, as it enables the rational design of peptides optimized for specific modes of action and informs combination strategies that leverage complementary anticancer pathways (12).

**Table 1:** Some ACP examples for the various modes of action.

Mode of action	Examples of peptides	Primary amino acid sequence	Peptide class	Net charge	Peptide source	References
Carpet	Human neutrophil peptides:					
	HNP-1	ACYCRIPACIAGERRYGTCIYQGRLWAFCC	β-sheet	+3	*Homo sapiens*	(95, 96)
	HNP-2	CYCRIPACIAGERRYGTCIYQGRLWAFCC				
	HNP-3	DCYCRIPACIAGERRYGTCIYQGRLWAFCC				
	Gomesin	QCRRLCYKQRCVTYCRGR	β-sheet	+6	Hemocytes of *Acanthoscurria gomesiana* spider	(97)
Barrel-stave	Aurein 1.2	GLFDIIKKIAESF	α-helix	+1	*Litoria raniformis*	(98)
	Melittin	GIGAVLKVLTTGLPALISWIKRKRQQ	α-helix	+6	Venom of the European honeybee *Apis Mellifera*	(99)
Combined carpet and barrel-stave	Citropin 1.1	GLFDVIKKVASVIGGL	α-helix	+2	*Litoria citropa* Frog	(100, 101)
	Gaegurin 5	FLGALFKVASKVLPSVKCAITKKC	α-helix	+4	*Rana Rugose* frog	(102, 103)
	Gaegurin 6	FLPLLAGLAANFLPTIICFISYKC				
Toroidal pore	Cecropin A Cecropin B	KWKLFKKIEKVGQNIRDGIIKAGPAVAVVGQATQIAK KWKVFKKIEKMGRNIRNGIVKAGPAIAVLGEAKAL	α-helix	+7	Silk moth *Hyalophora cecropia*	(104, 105)
				+8		
	Leucine leucine-37 (LL-37*)	LLGDFFRKSKEKIGKEFKRIVQRIKDFLRNLVPRTES	α-helix	+6	*Homo sapiens*	(106)
Combined toroidal pore and aggregate	Magainin 2	GIGKFLHSAKKFGKAFVGEIMNS	α-helix	+3	*Xenopus laevis* frog	(107)

#### 2.2.1 The carpet model

The carpet model mechanism resembles a detergent effect, where the ACPs accumulate electrostatically in a parallel fashion over the cell membrane, then get inserted into the hydrophobic core of the membrane lipid bilayer, redirecting the membrane phospholipids orientation, leading to its fluidity and micelle formation, and eventually formation of a gap within the cell membrane (11).

#### 2.2.2 The barrel-stave model

The ACP monomers are electrostatically attracted to the cell membrane, then they aggregate into a barrel shaped α-helical multimer, inserting their hydrophobic part into the hydrophobic core within the cell membrane, forming gaps within the membrane, through which the cell components leak out due to disrupted transmembrane potential and ion gradient (11).

#### 2.2.3 The toroidal pore model

First, ACPs accumulate parallel to the lipid bilayer until they reach a threshold concentration, after which they revert to their active form, realigning in a perpendicular position, inserting their hydrophilic part into the hydrophilic region of the membrane. This destabilizes the membrane, forming transmembrane toroidal pores, through which ACPs can get into the inner leaflet, disintegrate the pores, and reach the inner cell compartments, where they interfere with vital biochemical processes, such as DNA replication and protein synthesis (11, 13).

#### 2.2.4 The aggregate model

ACPs get electrostatically attracted to the cell membrane, where they combine with the lipid bilayer, resulting in micelle formation, which facilitates their non-specific insertion into the cell membrane, creating dynamic transmembrane pores. However, contrary to the Toroidal-pore model, they express no specific orientation (14, 15).

#### 2.2.5 Mechanistic integration and therapeutic implications

While the four models described above provide a framework for understanding ACP-membrane interactions, it is increasingly recognized that these mechanisms often coexist and may operate in concert. For instance, a peptide might initially disrupt membrane integrity through carpet-like accumulation before forming transient toroidal pores, or barrel-stave pores may evolve from aggregated peptide complexes. The predominant mechanism depends on multiple variables, including peptide concentration, amino acid sequence, membrane lipid composition, and the biophysical properties of the TME.

Of note, some ACPs may adopt more than one mode of action concurrently, e.g., Gaegurins. Surface-interaction activity of the ACP towards the cancer cell is enhanced by the peptide’s orientation angle, which destabilizes the cell membrane lipid packing, leading to its penetration.

This mechanistic understanding carries direct therapeutic relevance. First, knowledge of structure–function relationships allows researchers to engineer peptides that favor specific, more potent mechanisms of action. Second, peptides that employ multiple mechanisms simultaneously may exhibit synergistic anticancer effects while reducing the likelihood of resistance development. Third, understanding membrane interactions can guide the design of peptide-drug conjugates that combine membrane-disruptive activity with targeted delivery of chemotherapeutic agents. Finally, insights into mechanism-specific membrane requirements (e.g., lipid composition, charge density) can inform patient stratification strategies, potentially identifying tumors that are more likely to respond to ACP-based therapies.

## 3 Advances in AMPs as ACPs

Some AMPs can directly act as ACPs, both *in vitro* and *in vivo*, with selective cytotoxicity towards cancer cells. Drug-resistant cancer cells in particular possess highly negatively charged membranes, that can be targeted by the positive AMPs, which may either act directly to destroy the cancer cells (16), or enhance drug uptake through the disrupted membrane (17, 18).

Another application entails gene therapy through injecting AMPs or their gene precursors into the cancer cells. They can also be modified by tumor-targeting peptides to enhance their antitumor activity (19).

Modifications entailing combining AMPs with nanoparticles, can act through two possible ways: the nanoparticles function as drug-delivery systems for the AMPs (20, 21), or a modified AMP-nano-formulation functions all together as the drug carrier (22–24).

The Drug Bank Database has been mentioned to include 29 ACPs, some of which have been already approved by FDA and EMA, while others are still undergoing clinical trials for final approval (25). However, only 26 drugs are available with their data on the Drug Bank Database official website, and some of them, although mentioned in literature as approved by FDA and EMA, are reported through the Drug Bank Database as being not yet approved in the United States (US) or any other country. The information retrieved from PubChem and Drug Bank Data Base websites is based upon their own latest updates (Table 2).

Of note, while G17DT has shown promise in early clinical studies, including phase II trials that suggest potential efficacy, further investigation is needed to establish its effectiveness in advanced cases of gastric cancer. Nelipepimut-S (E75), a human epidermal growth factor receptor 2/neuroblastoma (HER2/neu) peptide vaccine, has demonstrated potential in early trials; however, results from phase III trials did not show a significant benefit in overall survival for the broader population, though some subgroup analyses indicated possible efficacy. It is crucial to interpret these results within the context of ongoing research, as many of these therapies are still under investigation and require further validation in larger, well-designed clinical trials.

**Table 2:** Drug Bank ACPs and their approval status.

FDA and EMA approval status	ACP drug name	Source structural information	Chemical structure	Target cancer studied	Countries of approval	References
	Drug bank accession number					
FDA and EMA approved	Buserelin DB06719	Synthetic primary amino acid sequence: XHWSYXLRP	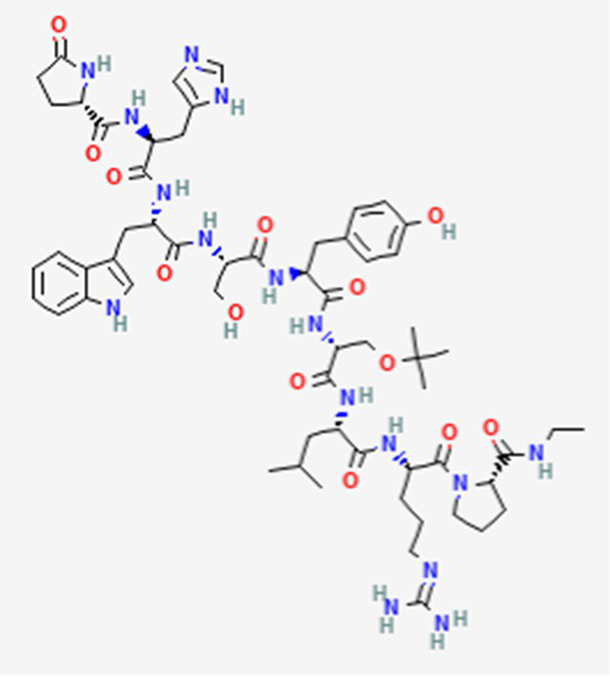	Reproductive organs, endometriosis and prostate cancer	Canada, UK, Ireland, Germany, Spain, Belgium, Luxembourg, Norway, Poland, Czech Republic, and other European countries. Japan, Singapore, Iran, Vietnam, Hong Kong. South Africa, New Zealand, and various countries in Latin America. Egypt, Tunisia, Lebanon	See text footnote 1, 2
	Dactinomycin DB00970	*Streptomyces parvullus*	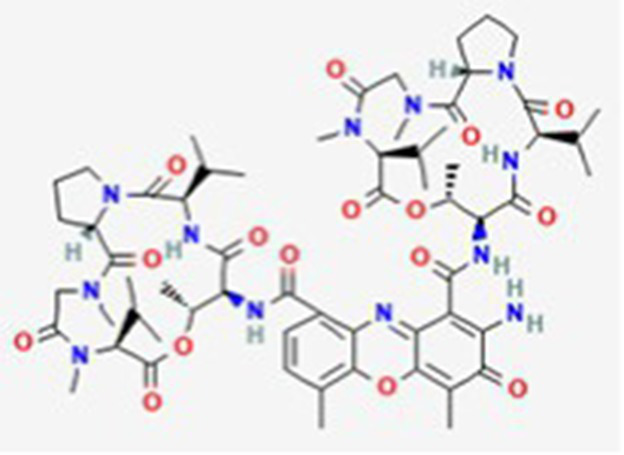	Childhood cancers such as Wilms tumor, Ewing sarcoma and rhabdomyosarco ma	US, Canada, UK, Germany, Finland, France, Greece, Ireland, Sweden, Australia, New Zealand Germany, Finland, France, Greece, Ireland, Sweden Argentina, Brazil, Chile, Colombia, Ecuador, Mexico, Peru, Venezuela, China, Japan, South Korea, India, Malaysia, Singapore, Thailand, Saudi Arabia, and UAE	See text footnote 3, 4, (27)
		Phenoxazine bound cyclic dipeptides				
	Plitidepsin (Aplidin) DB04977	Marine tunicate *Aplidium albicans* Depsipeptide	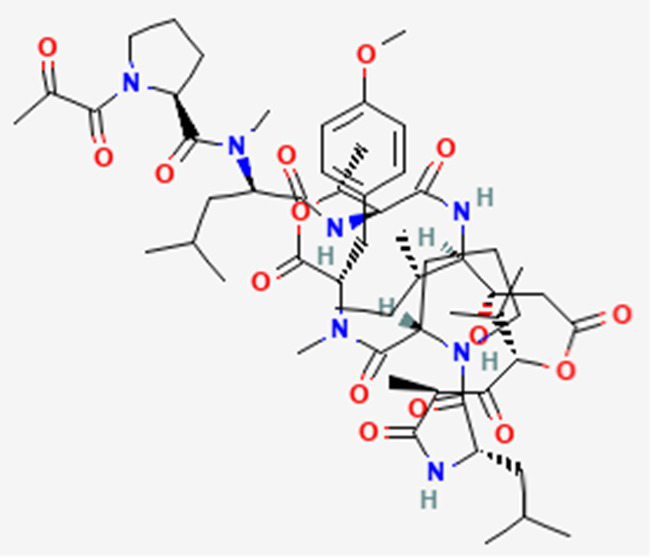	Multiple myeloma pancreatic, stomach, bladder and prostate cancers and some leukemias.	Australia, New Zealand, several Southeast Asian countries.	See text footnote 5, 6, (28, 29)
	Tebentafusp DB15283	Synthetic	Not available	Metastatic uveal melanoma.	US, Canada, all EU member states, Iceland, Liechtenstein, Norway, Australia, Israel, Spain, Bulgaria, Czech Republic, and Lithuania	See text footnote 7, (30, 31)
	Triptorelin DB06825	Synthetic primary amino acid sequence: XHWSYWLRPG	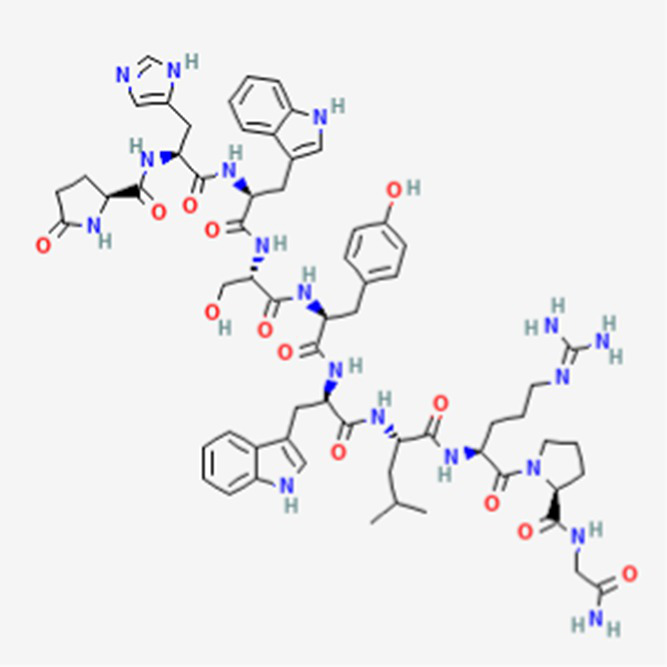	End-stage prostate cancer.	US, Canada, UK, France, Germany, UK, Austria, Denmark, Finland, Norway, Sweden, Poland, Slovakia, Czech Republic, Brazil, Mexico, and Peru.	See text footnote 8, 9, (32)
Pending approval	ATN-161 DB05491	Synthetic primary amino acid sequence: PHSCN	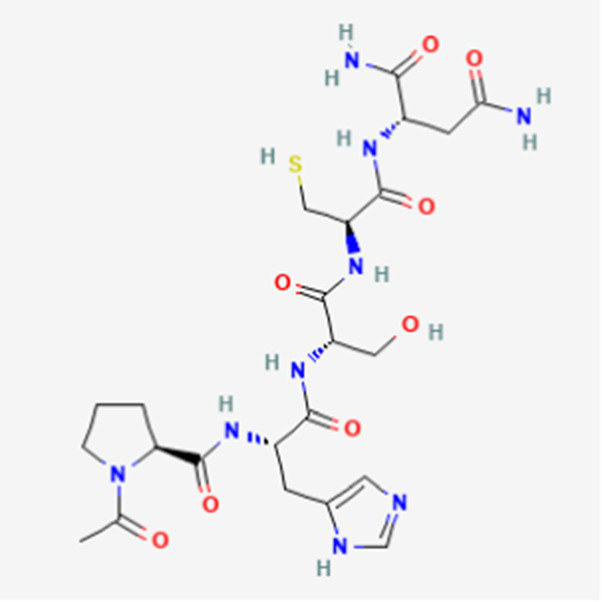	Brain cancer, breast cancer and other unspecified cancers.		See text footnote 10, 11, (33)
	Canfosfamide DB04972	Synthetic prodrug	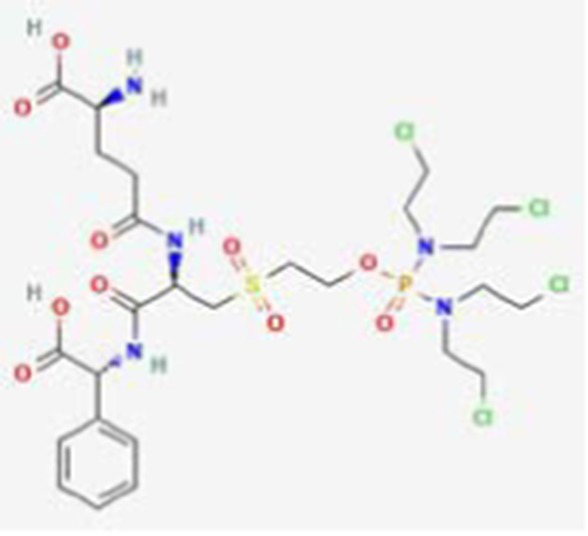	Chemotherapy-resistant ovarian cancer		See text footnote 12, 13, (34, 35)
	CTCE-0214 DB05934	Synthetic primary amino acid sequence: KPVSLSYRAPFRFFG GGGLKWIQEYLEKA LN	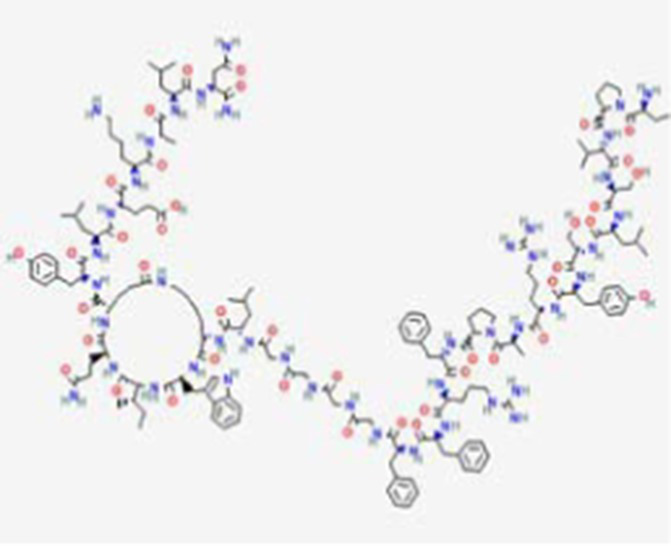	Suppression of plasma TNF-α in acute endotoxemia and zymosan-induced multiple organ dysfunction syndrome, macrophage associated lung cancer, and unspecified cancers.		See text footnote 14, 15, (36, 37)
	Darinaparsin DB06179	Metabolic intermediate of inorganic arsenicals (iAs) *in vivo*	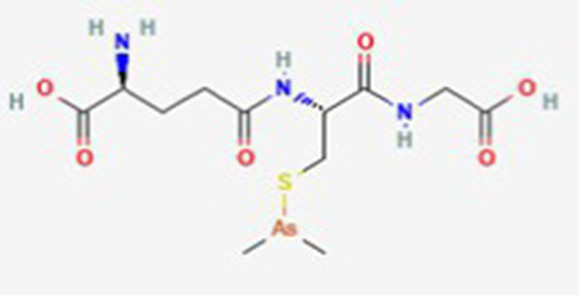	Refractory peripheral T-cell lymphoma, solid tumors, multiple myeloma, and liver cancer		See text footnote 16, 17, (38, 39)
		Primary amino acid sequence: XXG				
	G17DT	Gastrin-17 immunogen	Not available	Gastrointestinal tract cancers		(40–43)
	No accession number	Nonapeptide derived from the amino-terminal of human gastrin-17 (pyroEGPWLEEEEEAYGWMDF-NH_2_) linked to diphtheria toxoid				
	IRL-1620 DB06138	Synthetic primary amino acid sequence: DEEAVYFAHLDIIW	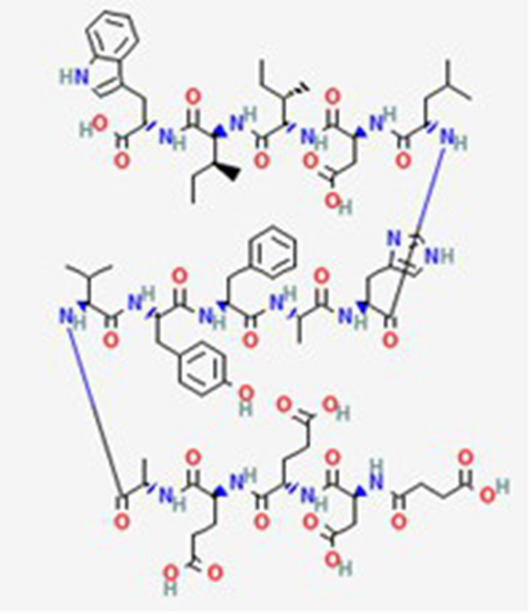	Neurologic tumors.		See text footnote 18, 19, (44)
	Iseganan DB16010	Synthetic primary amino acid sequence: RGGLCYCRGRFCVC VGR	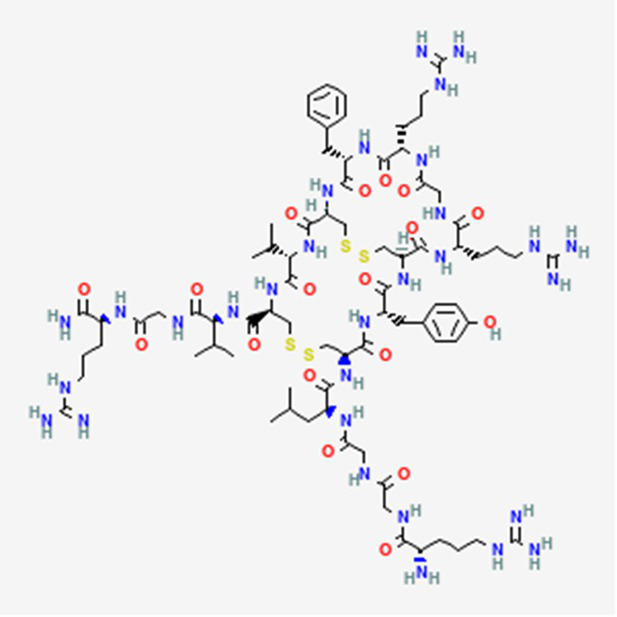	Reduces oral mucositis in patients undergoing chemotherapy or radiotherapy for head and neck cancers.		See text footnote 20, 21, (45, 46)
	Labradimil DB06549	Synthetic nine-amino acid peptide (no sequence available on PubChem)	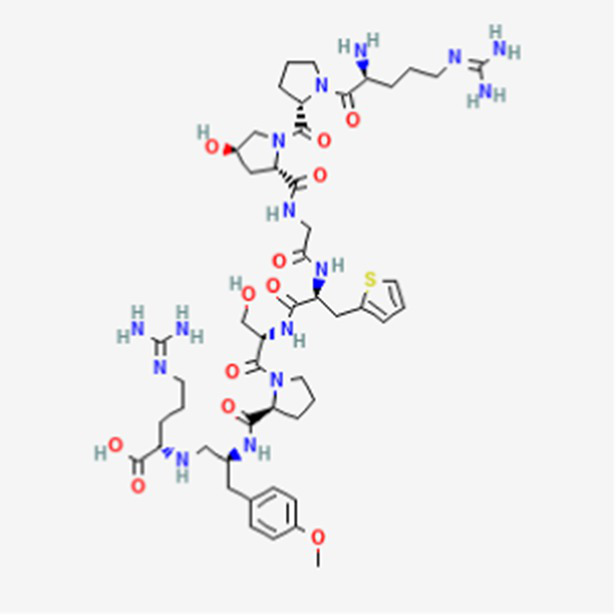	Brain cancer.		See text footnote 22, 23, (47)
	Nelipepimut-S DB06226	Synthetic vaccine primary amino acid sequence: KIFGSLAFL	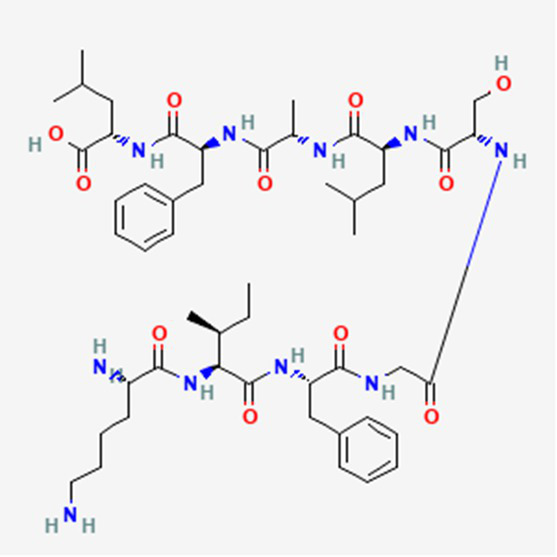	Reduces cancer recurrence and prolongs survival in cancer patients with tumors that express the HER2/neu oncoprotein.		See text footnote 24, 25, (48)
	PM02734 DB05158	Synthetic derivative of the depsipeptide Kahalalide F found in the marine mollusk *Elysia rufescens*	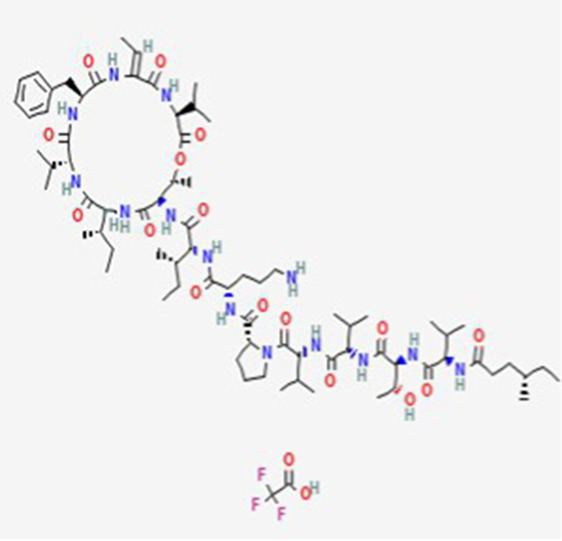	Wide range antiproliferative activity, including colon, prostate, breast and lung cancers.		See text footnote 26, 27, (49–51)
	SF1126 DB05210	Synthetic RGDS-conjugated proagent of LY294002	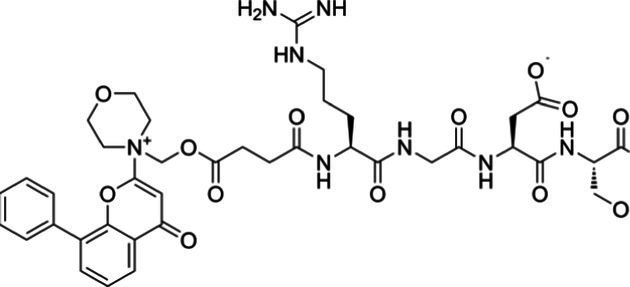	Metastatic squamous neck carcinoma, neuroblastoma, advanced hepatocellular carcinoma and Ewing sarcoma.		See text footnote 28, 29, (52)
	Teverelix No accession number	Synthetic primary amino acid sequence: GPRLGYSWHE	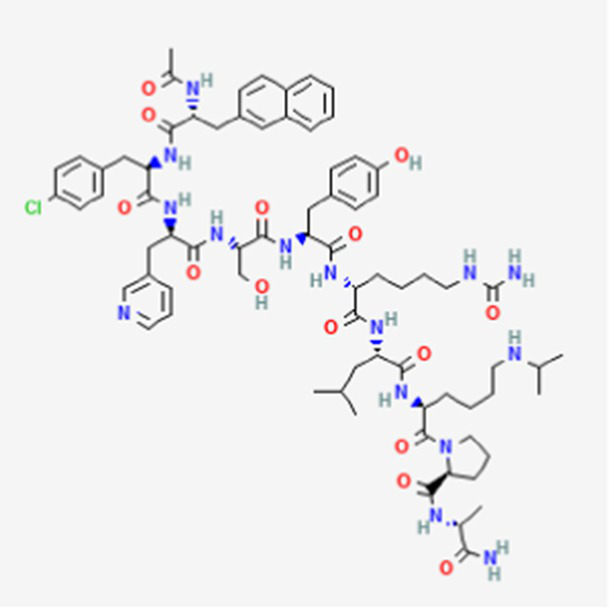	Prostate cancer		See text footnote 30, 31, (53)
	Tigapotide DB04985	Synthetic primary amino acid sequence: EWQTDNXETXTXYE T	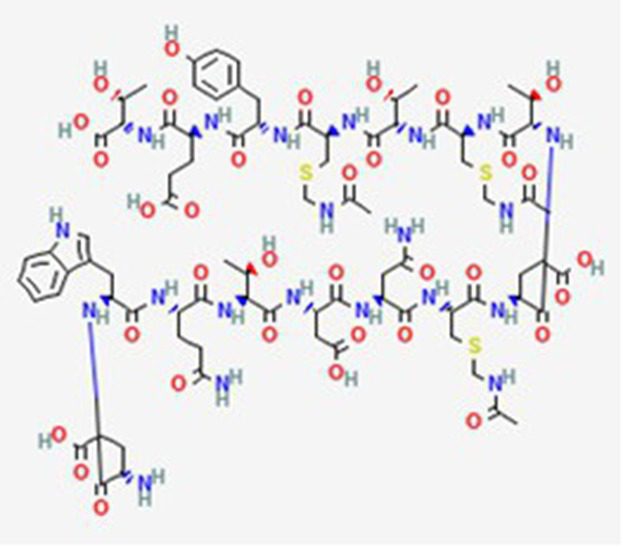	Advanced prostate cancer.		See text footnote 32, 33
Trial drugs	Balixafortide DB15370	Synthetic	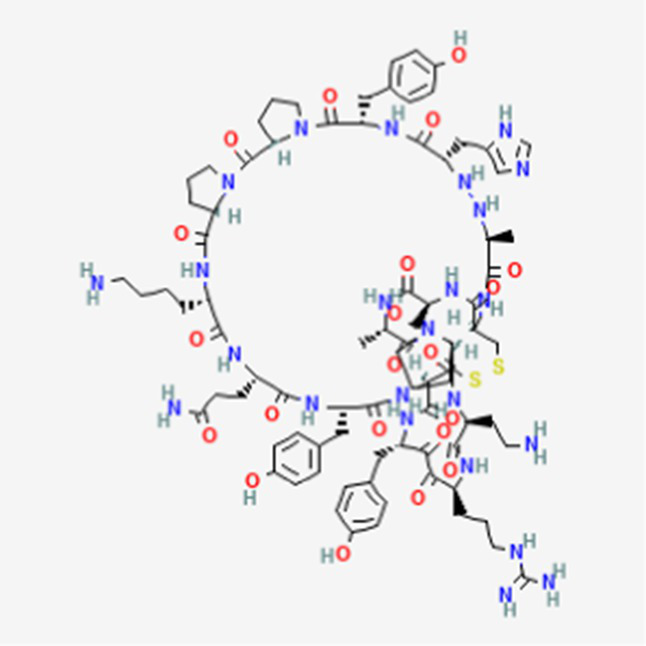	Breast cancer		See text footnote 34, 35, (54)
	Bleomycin A6 (Boanmycin) DB12992	*Streptomyces verticillus Glycoprotein*	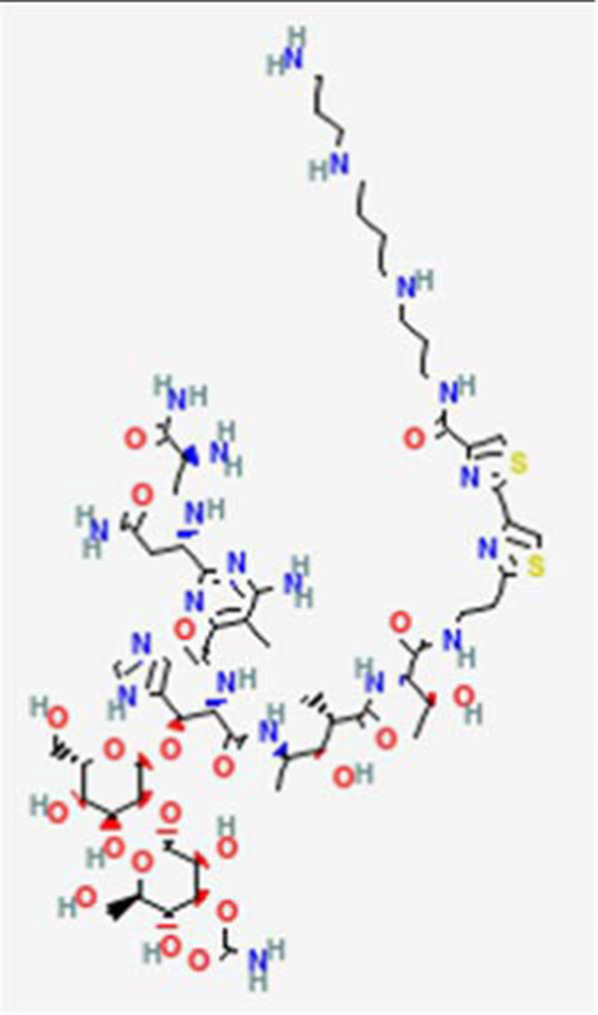	Squamous cell lung cancer, esophageal cancer, hepatocellular carcinoma and hepatic metastasis, colon and colorectal cancer, multiple myeloma, and cervical cancer (*in vitro*).		See text footnote 36, 37, (55–57)
	Bombesin DB11724	Amphibian (frog skin) *Bombina bombina Bombina variegata variegata*	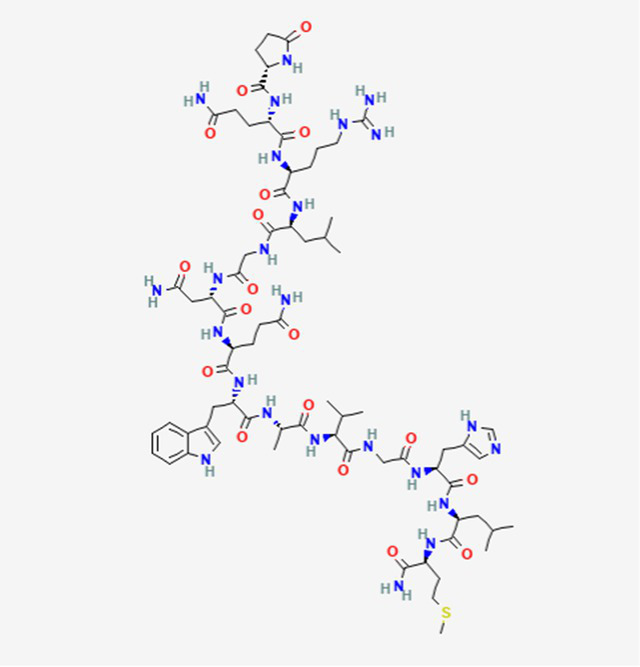	Prostate cancer and small cell lung cancer.		See text footnote 38, 39, (58, 59)
		Primary amino acid sequence: XQRLGNQWAVGHL M				
	Dolastatin 10 DB12730	Sea hare *Dolabella auricularia*	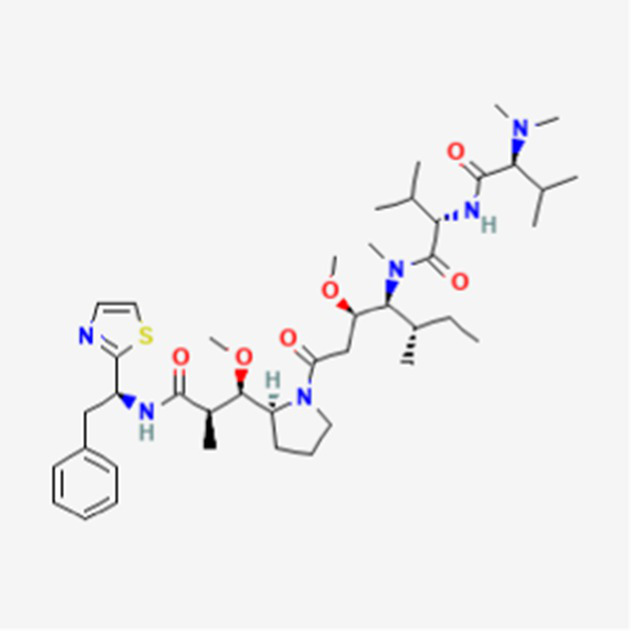	Leukemia, small and large cell lung cancer, prostate cancer, ovarian adenocarcinoma, brain glioma, renal carcinoma, colon carcinoma and melanoma.		See text footnote 40, 41, (60, 61)
		Tetrapeptide: (S)-dolavaline (Dov, 2) /(S)-valine (Val, 3)/(3R,4S,5S)-dolaisoleuine (Dil, 4)/(2R,3R,4S)-dolaproine (Dap, 5)/(S)-dolaphenine (Doe, 6), and a primary amine, possibly originating from phenylalanine, serving as carboxyl C-terminus.				
	Soblidotin DB12677	Marine bacterium *Salinispora tropica*	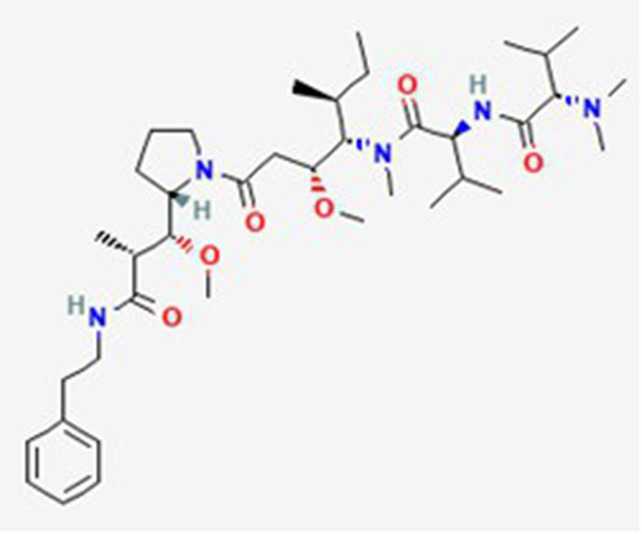	Sarcoma, lung cancer and unspecified adult solid tumor.		See text footnote 42, 43, (62)
		Tetrapeptide analogue of dolastatin 10				
	LTX-315 (Ruxotemitide) DB12748	Synthetic human lactoferrin derivative	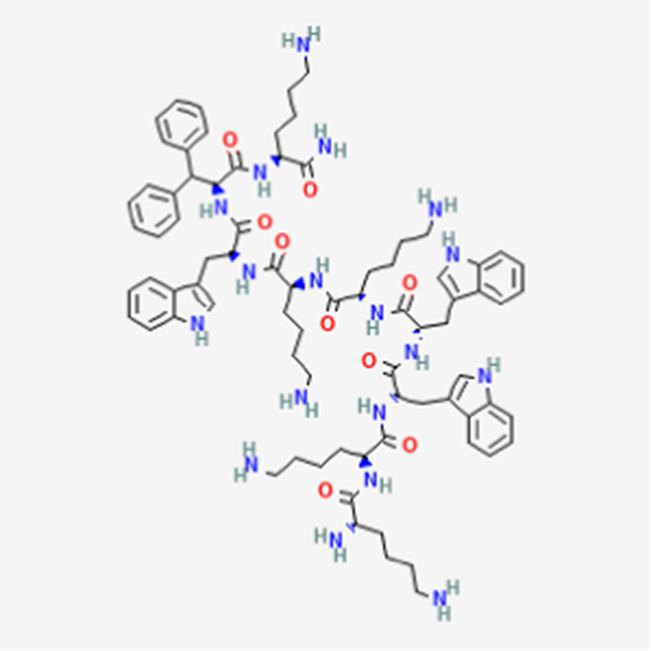	Lymphoma, melanoma, and breast cancer.		See text footnote 44, 45, (63)
		Primary amino acid sequence: KKWWKKWXK				
	Ozarelix DB12581	Synthetic primary amino acid sequence: XXXSYXXRPA	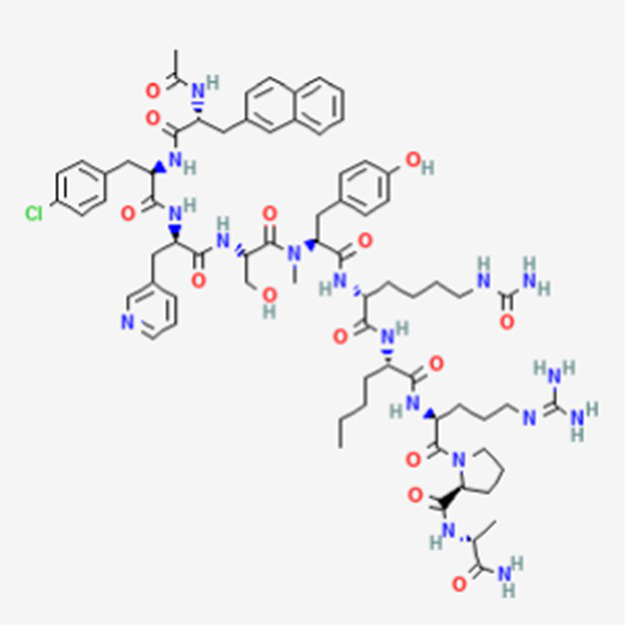	Prostate cancer		See text footnote 46, 47, (64)
	TAK-448 DB11975	Synthetic analogue of kisspeptin	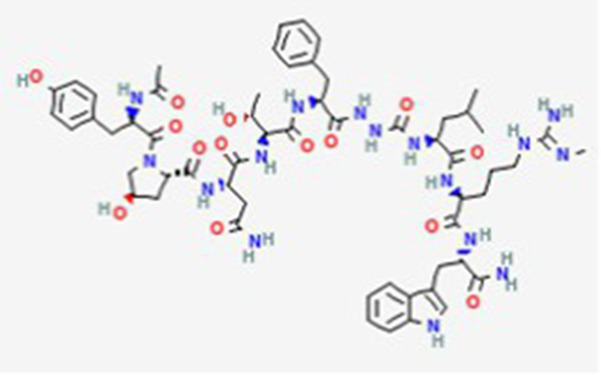	Prostate cancer		See text footnote 48, 49, (65)
	Valspodar DB11869	Synthetic homodetic analogue of cyclosporin-A	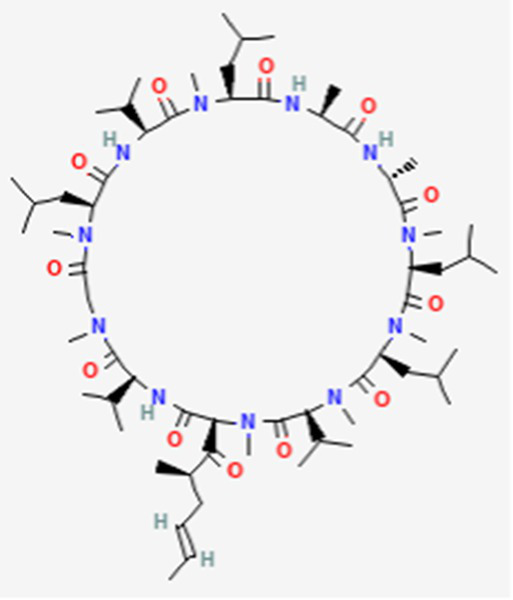	Sarcoma, leukemia, lymphoma and breast cancer.		See text footnote 50, 51, (66)
	VEGFR2–169 (Elpamotide) DB16152	Synthetic peptide vaccine	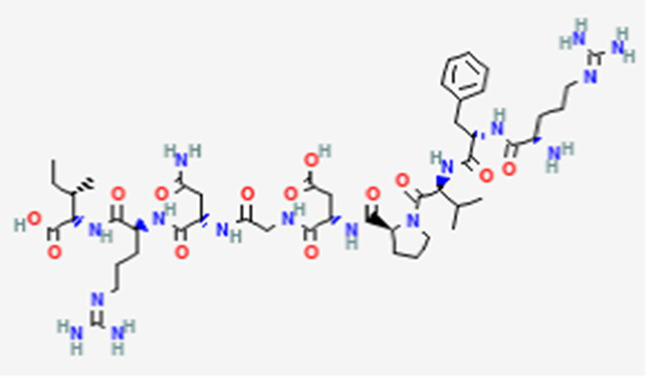	Pancreatic cancer		See text footnote 52, 53, (67)
		Primary amino acid sequence: RFVPDGNRI				
	Zoptarelin doxorubicin DB12755	Synthetic	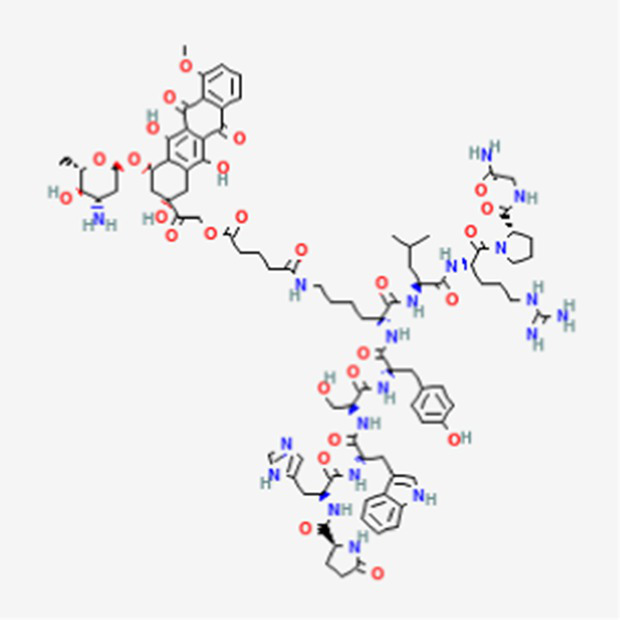	Breast cancer, ovarian cancer, prostate cancer, endometrial cancer, and urothelial carcinoma.		See text footnote 54, 55, (68)

One of the most critical issues of cancer is heterogeneity. Carcinogenesis-inducing mutations often target molecules at the cell surface, eliciting more or less random perturbations in their structures and functions, notably those related to immune defense, ion channels, transporters, and biochemical signaling. The result is chaos in the signals the cell emits and receives and differential changes in interaction with the TME. The immediate consequence is the heterogeneity in cancers, an insurmountable and critical issue. AMPs readily bypass the defects in the plasma membrane to which they bind via electrostatic forces, and permeate the lipid bilayer thanks to the prevalence of hydrophobic amino acids, to access the cell interior and express their impact.

### 3.1 ACPs approved by FDA and EMA mechanism of action

#### 3.1.1 Buserelin

This peptide is approved in some countries, but not in the US. It is a synthetic peptide, analogous to the luteinizing hormone-releasing hormone (LHRH) agonist^1^^,^^2^. It desensitizes the pituitary gland gonadotrophin-releasing hormone receptor (GnRHR), reducing gonadotropin levels, thereby reducing the synthesis and release of testosterone in males, and inhibiting estrogen secretion in females (26).

#### 3.1.2 Dactinomycin

It is composed of two cyclic peptides joined to a phenoxazine, and is approved in the US and other countries. It is an actinomycin that acts as a DNA adduct. Its strong, yet reversible binding to DNA inhibits the RNA polymerase elongation step, thereby inhibiting protein synthesis.^3^^,^^4^ It is used to treat a wide variety of cancers, including childhood cancers (27).

#### 3.1.3 Plitidepsin

This drug is a cyclic depsipeptide since it includes also ester bonds (Table 2). It has not yet been approved in the US or other countries.^5^^,^^6^ It has been isolated from the marine tunicate *Aplidium albicans* (28). It acts through cell growth inhibition and induction of apoptosis, through targeting the elongation factor 1A2 (eEF1A2) in eukaryotes (29), supposedly through multiple modes of action that are not yet fully elucidated (see text footnote 5).

#### 3.1.4 Tebentafusp

This synthetic drug has been approved for use in the US and other countries. It has a novel immunotherapeutic mechanism of action for immune-mobilizing monoclonal T cell receptors against cancer (ImmTACs). ImmTACs bind to target cancer cells that express a specific targeted antigen, thereby recruiting cytotoxic T cells to lyse the cancer cells, such as melanocytes. Tebentafusp is a bispecific glycoprotein100 (Gp100) Peptide-HLA-directed CD3 T Cell engager, where Gp100 is a transmembrane glycoprotein, highly expressed in melanoma cells, but not in normal melanocytes or tissues^7^ (30, 31).

#### 3.1.5 Triptorelin

This is a synthetic decapeptide, that has been approved in the US and other countries. It is a LHRH agonist, but with higher potency due to a structural modification with D-tryptophan substitution at position 6, which enhances its receptor binding affinity, thereby providing more potent GnRHR activation and longer half-life in plasma compared to native GnRH. It reversibly represses the secretion of gonadotropin, and has been proven to be effective in end-stage prostate cancer. However, further research is recommended to mitigate its potential clinical adverse effects (32).^8^^,^^9^

### 3.2 ACPs pending approval by FDA and EMA mechanism of action

#### 3.2.1 ATN-161

This synthetic pentapeptide is a non-RGD (arginylglycylaspartic acid) based integrin-binding peptide, that targets alpha-5 beta-1 and alpha-v beta-3 domains, thereby inhibiting the migration and adhesion of specific integrins on activated endothelial cells crucial for tumor angiogenesis.^10^ It has been reported to block breast cancer and hepatocellular carcinoma growth and metastasis through downregulation of EMP2 transmembrane protein, which is crucial for cell surface molecule binding, transmembrane transport and cell adhesion and metastasis (33).^11^

#### 3.2.2 Canfosfamide

This is a synthetic oligopeptide drug that acts as a modulator for the enzyme glutathione S-transferase P1-1 (GST P1-1), which is overexpressed in various human cancer cells, and its high levels have been correlated with a poor prognosis and developing resistance to some chemotherapies. GST P1-1 splits canfosfamide into two active fragments: a glutathione analog fragment that remains bound to the enzyme, thereby limiting its inactivation effect on drugs, and an active cytotoxic fragment that reacts with RNA, DNA and proteins, resulting in cancer cell death.^12^^,^^13^ (34) However, its efficacy on ovarian cancer is recommended to be further investigated (35).

#### 3.2.3 CTCE-0214

This synthetic peptide is an analog for stromal cell-derived factor (SDF-1*α*), and agonist of chemokine CXC receptor 4 (CXCR4). It increases the levels of white blood cells, platelets and stem cells. *In vivo* trials in murine models showed it suppressed plasma tumor necrosis factor-alpha (TNF-α) increases in acute endotoxemia and following zymosan-induced multiple organ dysfunction syndrome that could lead to increased risk of carcinogenesis (36).^14^^,^^15^ It has been also reported to regulate macrophages associated with lung cancer (37).

#### 3.2.4 Darinaparsin

This is a novel organoarsenic mitochondrial-targeted drug that has been approved in Japan for the treatment of refractory peripheral T-cell lymphoma. It is composed of dimethylarsenic conjugated to glutathione, and is a metabolite of inorganic arsenic (AsIII) during its biotransformation in the body (38).^16^^,^^17^ Studies on its effect on human acute promyelocytic leukemia cell line (NB4) indicate a convergence of both the intrinsic and extrinsic apoptotic pathways, associated with DNA damage. It is also suggested that it triggers oxidative stress, which contributes primarily to apoptosis induction (39).

#### 3.2.5 G17DT

G17DT is a vaccine against gastrin-17 hormone that promotes the growth of a number of gastrointestinal tract cancers. It has been reported to be effective and well tolerated in the treatment of advanced cases (40). It is composed of a nonapeptide derived from the amino-terminal of human gastrin-17 (pyroEGPWLEEEEEAYGWMDF-NH_2_) linked to diphtheria toxoid (a 535 amino acid-polypeptide chain of two subunits linked together by disulfide bridges) (41–43). Clinical evaluation of G17DT has included phase II studies in advanced gastric, pancreatic, and colorectal cancers, where the vaccine demonstrated an acceptable safety profile and showed evidence of biological activity, including the induction of anti-gastrin antibodies and trends toward improved survival in some patient subgroups (40). Despite this early promise, the vaccine has not yet progressed to regulatory approval, and its efficacy remains to be confirmed in adequately powered phase III trials. As with many cancer vaccines, patient selection and immune response heterogeneity appear to influence outcomes, highlighting the need for predictive biomarkers in future studies.

#### 3.2.6 IRL-1620

It is a synthetic peptide analogue of endothelin-1, that acts as a highly selective agonist for endothelin B receptor on the endothelial-rich lining of the blood vessels characteristic of growing tumors, thereby enhancing blood flow and drug delivery to the tumor, without affecting the smooth muscle-lining blood vessels of normal tissues (44).^18^^,^^19^

#### 3.2.7 Iseganan

It is an analog of protegrin-1 (PG-1) AMP that has a membrane-binding capacity, found in pig leukocytes, possessing broad spectrum antimicrobial effect against Gram positive and Gram-negative bacteria, yeast and fungi. It lowers the risk of developing oral mucositis in patients undergoing chemotherapy or radiotherapy for head and neck cancers, through reducing the local microflora density. Recent studies on peptides modified from iseganan achieved more desirable antibacterial activities, not relying on synergy with conventional antibiotics (45, 46).^20^^,^^21^

#### 3.2.8 roadmaLabradimil

It is a bradykinin analog that targets the B2 bradykinin receptor.^22^^,^^23^ Studies on animals have shown its ability as a vasoactive peptide to transiently disrupt the blood–brain barrier, allowing delivery of chemotherapy drugs to the brain (47).

#### 3.2.9 Nelipepimut-S

It is HER2/neu peptide-based T-cell immunotherapy, that prevents cancer recurrence and prolongs survival in cancer patients, with tumors expressing the HER2/neu oncoprotein, through inducing a sustained antigen-specific T-cell response. It targets and inhibits Receptor tyrosine-protein kinase erbB-2 and HLA class I histocompatibility antigen, A alpha chain (HLA-A) (48)^24^^,^^25^ Early-phase trials demonstrated immunogenicity and suggested potential clinical benefit, including reduced recurrence rates in node-negative and node-positive breast cancer patients, (see text footnote 24) prompting phase III evaluation; however, the large, randomized trial in breast cancer patients with low-to-intermediate HER2 expression did not show a statistically significant improvement in disease-free survival for the vaccine-treated group compared to placebo (see text footnote 25). Subsequent analyses suggested that certain subgroups, such as patients with triple-negative breast cancer or those who mounted robust immune responses, may derive greater benefit, though these findings require prospective validation (48).

The trajectory of Nelipepimut-S highlights critical considerations for cancer vaccine development: the importance of optimal patient selection, the need for reliable immune monitoring, and the potential value of combination strategies to overcome tumor-mediated immunosuppression. Ongoing research continues to explore its utility in defined patient populations and in combination with other immunotherapies.

#### 3.2.10 PM02734

It is a trifluoroacetate synthetic derivative of the depsipeptide Kahalalide F, originally isolated from the marine mollusk *Elysia rufescens*, as the natural peptide cannot be isolated in enough amounts (49).^26^^,^^27^ It has wide antiproliferative effect against various tumors, including colon, pancreas, lung, breast and prostate cancers (see text footnote 27). Kahalalide F has demonstrated necrosis-like cell death in the tested cell lines, with suggested involvement of the mitochondria and lysosomes in the mechanism (50). PM02734 has been reported to inhibit gene expression for certain genes involved in DNA replication, as well as in tumor cell proliferation and growth (51).

#### 3.2.11 SF1126

It is an RGDS-conjugated pro-agent of the protein inhibitor LY294002, which inhibits inhibiting phosphoinositide 3-kinase enzymes involved in growth control and initiation of translation. It exhibits high solubility, thereby can be administered through injections. It binds to specific integrins within the tumor compartment and induces cell apoptosis. Clinical trials showed its efficacy in treating metastatic squamous neck carcinoma, neuroblastoma and advanced hepatocellular carcinoma. It has also demonstrated benefit in phosphoinositide-3-kinase inhibition in Ewing sarcoma, the second occurring pediatric bone cancer (52).^28^^,^^29^

#### 3.2.12 Teverelix

It is a GnRH agonist water-soluble decapeptide, that shows reversible competitive binding to GnRHR,^30^ resulting in suppression of testosterone, prostate-specific antigen, follicle-stimulating hormone, and luteinizing hormone in cases with prostatic hyperplasia and cancer, and endometriosis, with a good safety profile (53).^31^

#### 3.2.13 Tigapotide

It is a 15-mer peptide, derived from the prostate secretory protein PSP94, which is one among three predominant proteins found in the human seminal fluid, and is down regulated in advanced prostate cancer. It has been suggested to act through multiway signal transduction inhibition, including apoptosis, anti-angiogenesis and anti-metastasis.^32^^,^^33^

### 3.3 ACPs under trial mechanism of action

#### 3.3.1 Balixafortide

It is a CXCR4 selective antagonist,^34^^,^^35^ with high potency in reducing myeloid cells immunosuppression in cases of breast cancer (54).

#### 3.3.2 Bleomycin A6

It is a glycoprotein antibiotic originally produced by the bacterium *Streptomyces verticillus*. It has been shown to induce DNA breakage, as well as changes in the TME. Studies have reported its role in treating a variety of conditions, including squamous cell lung cancer, esophageal cancer, hepatocellular carcinoma and hepatic metastasis, colon and colorectal cancer (55).^36^^,^^37^ It has also demonstrated efficacy in overcoming anticancer drug-developed resistance in multiple myeloma, damaging cancer cell DNA and functionally impairing the endoplasmic reticulum (56). Bleomycin-loaded cinnamon oil nanoemulsion has demonstrated higher apoptotic activity *in vitro* on HeLa cervical cancer cells (57).

#### 3.3.3 Bombesin

It was first isolated from the skin of the European frogs *Bombina bombina* and *Bombina* var*iegata variegate* (58). Bombesin receptors are overexpressed in various tumor cells. The peptide agonist activities in the human body include Bombesin receptor subtype-3, Neuromedin-B receptor and Gastrin-releasing peptide receptor, and has shown promising results in treating various forms or prostate cancer.^38^^,^^39^ Chemotherapeutic agents conjugated with bombesin have shown efficacy in overcoming drug-resistance in small cell lung cancer (59).

#### 3.3.4 Dolastatin 10

It is a tetrapeptide marine metabolite isolated from the sea hare *Dolabella auricularia*, that acts as antineoplastic agent, apoptosis inducer and a microtubule-destabilizing agent, inhibiting human tubulin beta-2A chain. It is a 1,3-thiazole and its activity is attributed to L-valine residue.^40^^,^^41^ It has demonstrated *in vitro* more potent lethal effects than most anticancer drugs, on various cancer cell types, including leukemia, small and large cell lung cancer, prostate cancer, ovarian adenocarcinoma, brain glioma, renal carcinoma, colon carcinoma and melanoma (60, 61).

#### 3.3.5 Soblidotin

It is a dolastatin 10 analogue, having the terminal dolaphenine amino-acid residue in dolastatin replaced by phenylamine group. Similar to dolastatin, it also acts as a tubulin polymerization inhibitor, and showing also antineoplastic activity and apoptosis induction through cell cycle arrest. Its activity is attributed to 2-phenylethylamine and L-valine residues. It has been studied in cases of sarcoma, lung cancer, and unspecified adult solid tumor (62).^42^^,^^43^

#### 3.3.6 LTX-315

It plays a dual role of direct lysis of the cancer cells, and immunostimulatory effect. Direct transdermal injection into the tumor leads to lysis of the cell membrane lysis and permeabilization of the mitochondrial membrane. On the other hand, innate and adaptive immune responses, mediated by anti-tumor natural killer (NK) cells, cytotoxic T lymphocytes, and natural killer T (NKT) cells can be triggered by exposure of the tumor antigens to the immune system. This may even trigger an immune response against tumor associated antigens on distant tumors from the primary tumor. It has been tested on various cancers, including lymphoma, melanoma and breast cancer (63).^44^^,^^45^

#### 3.3.7 Ozarelix

It is a GnRHR antagonist, that inhibits gonadotrophins secretion, thereby decreases the risk of hormone-dependent diseases including prostate cancer. Studies on two androgen independent prostate cancer cell lines: DU145 PC3 showed its ability to sensitize both cell lines to the membrane protein TNF-related apoptosis-inducing ligand (TRAIL), which initially induces apoptosis through its binding to the death receptors DR4 and DR 5 located on the cell surface. It has been investigated in prostate cancer (64).^46^^,^^47^

#### 3.3.8 Tak-448

It is a more potent analogue of the natural 54 amino acid peptide kisspeptin, encoded by the human Kiss-1 gene, and purified from human placenta, with improved *in vivo* biological stability. Both the analogue and the natural peptide act as modulators of the G-coupled metastasis suppressor Kiss-1 receptor. The peptide/receptor system is a key regulator of the hypothalamic–pituitary-gonadal axis, that stimulates the release of gonadotrophins.it has been applied in the treatment of prostate cancer (65, 66).^48^^,^^49^^,^^50^^,^^51^

#### 3.3.9 Valspodar

It is a tumor necrosisnon-toxic cyclosporin-A analogue that inhibits p-glycoprotein, thereby restoring drug activity in drug-resistant advanced solid tumor cells. Its anti-tumor activity also entails caspase-mediated apoptosis. It has been investigated in cases of sarcoma, leukemia, lymphoma and breast cancer (66) (see text footnote 51).

#### 3.3.10 VEGFR2–169

It is a peptide vaccine, including an epitope of the vascular endothelial growth factor receptor 2 (VEGFR2). This receptor plays a key role in promoting angiogenesis in cancer cells and thereby enhancing cell proliferation. Drugs that target VEGFR2 are developing drug resistance over the course of chemotherapy. VEGFR2-169 stimulates a cytotoxic T lymphocyte response against VEGFR2-expressing tumor cells. It has been investigated in pancreatic cancer (67).^52^^,^^53^

#### 3.3.11 Zoptarelin doxorubicin

It is an agonist of the GnRH-1 receptor, conjugated to doxorubicin, which is an anthracycline antibiotic. The peptide-drug combination binds to the highly expressed GnRH-1 receptor on ovarian and endometrial cell membranes, and penetrates into the cells, where the doxorubicin moiety intercalates into DNA, inhibiting topoisomerase II, thereby preventing DNA replication and cell proliferation. The peptide-drug combination has shown less toxicity than doxorubicin alone. It has been investigated in cases of breast cancer, ovarian cancer, prostate cancer, endometrial cancer, and urothelial carcinoma (68).^54^^,^^55^

**Figure 2:**
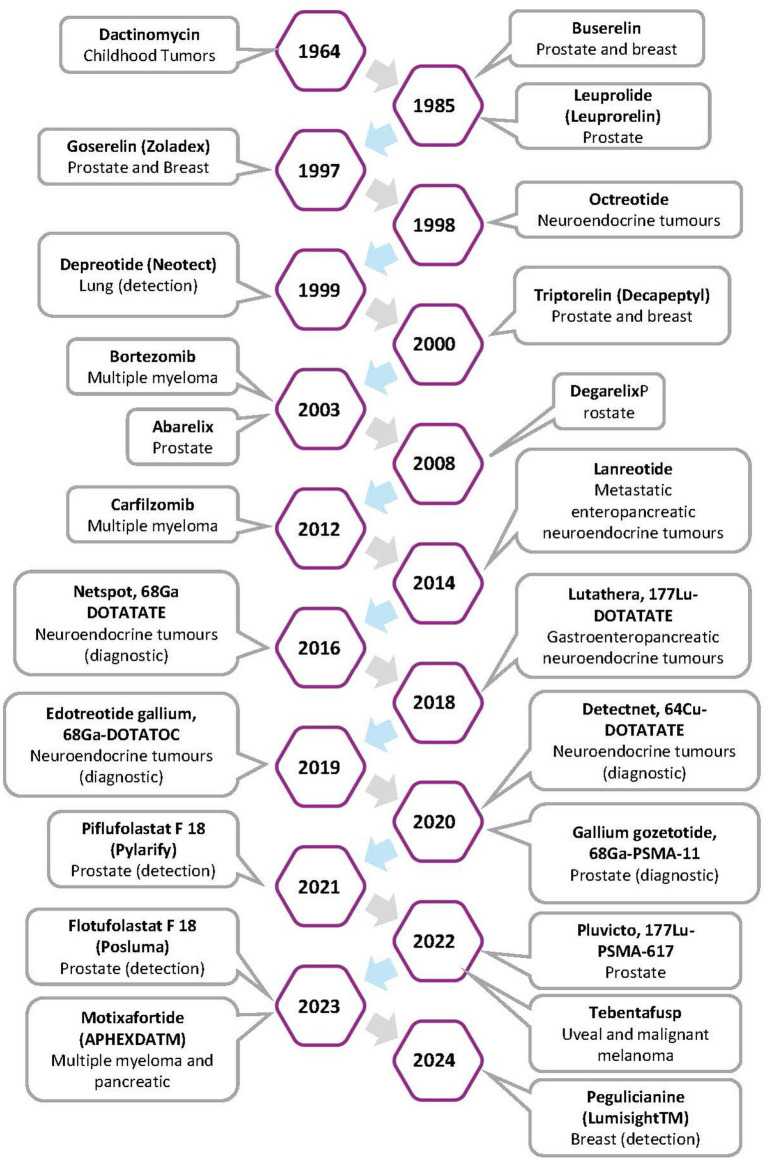
Timeline of approved ACPs and their target cancer.

Future in-depth studies are still required to elucidate ACPs mechanisms, correlating their sensitivity both *in vitro* and *in vivo*, discern their liability to enzymatic degradation and hydrolysis by serum proteins, maintain their anti-cancer effect, while reducing any potential hemolytic toxicity (11, 24) (see Figure 2).

## 4 Immunomodulatory role of AMPs in the TME

**Figure 3:**
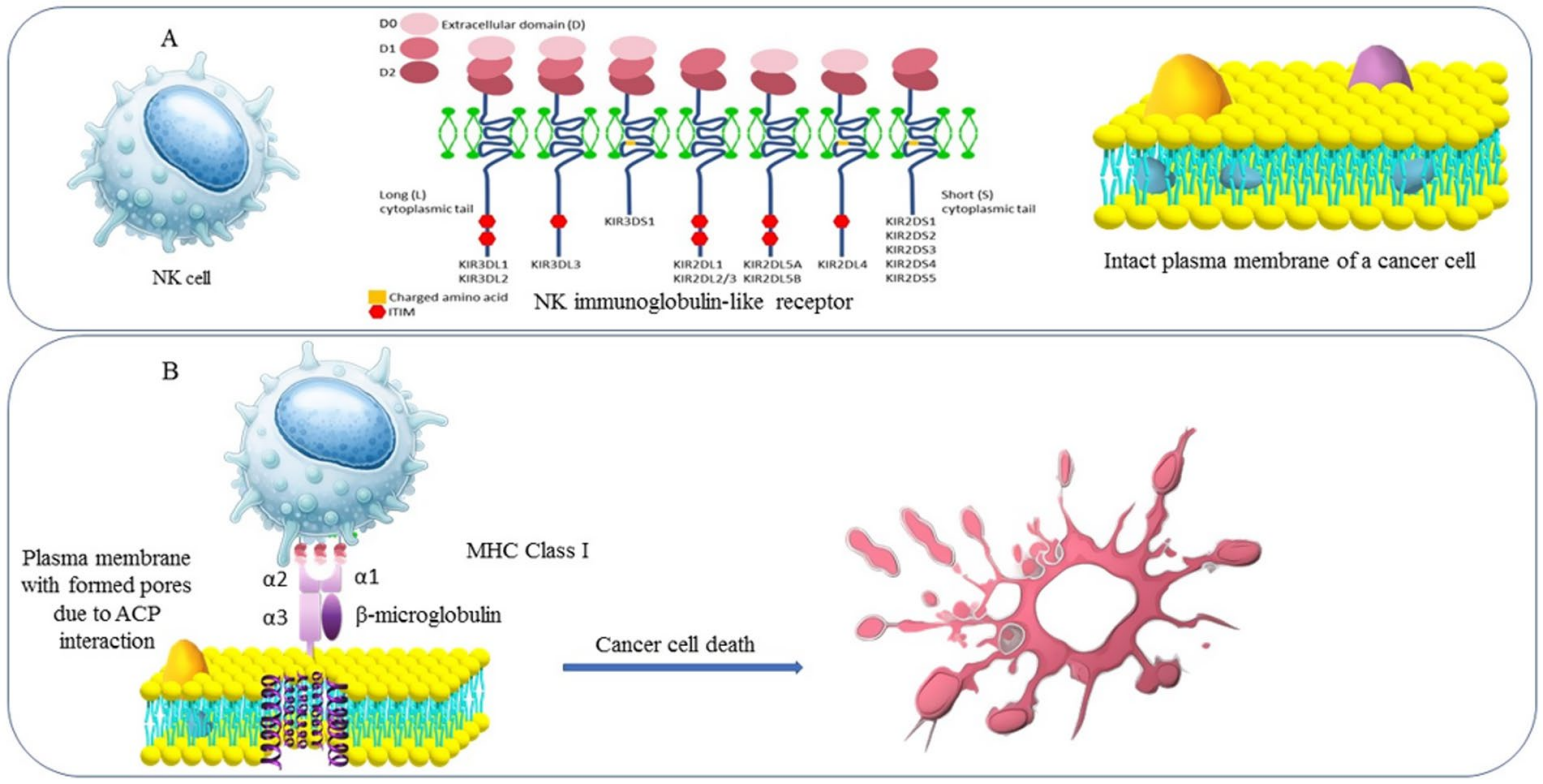
**(a)** NK cells and their immunoglobulin-like receptor unbound to the intact cancer cell membrane. **(b)** NK cells and their immunoglobulin-like bind to MHC class I exposed within the pores formed by ACP resulting in cancer cell destruction.

The immune system plays a critical role in surveilling and eliminating malignant cells through the coordinated action of innate and adaptive immunity. The innate immune system serves as the first line of defence, reacting rapidly to aberrant cells without prior antigen exposure by using the likes of macrophages, neutrophils, dendritic cells, NK cells, and others (Figures 3a,b). In cancer, innate cells can recognize and attack tumors through phagocytosis, cytotoxic granule release, and inflammatory cytokine production (69). The adaptive immune system, involving T lymphocytes (CD4^+^ helper and CD8^+^ cytotoxic T cells) and B lymphocytes, provides antigen-specific and memory-driven responses. Activation depends on antigen presentation, primarily by dendritic cells, which prime T cells to recognize and lyse cancer cells. However, tumors often evade immune detection through mechanisms such as downregulation of antigen presentation, expression of immune checkpoint molecules like Programmed Death-Ligand 1 (PD-L1), and establishment of an immunosuppressive microenvironment. Overcoming these barriers is central to CI.

In this context, AMPs have emerged as multifaceted immunomodulators within cancer immunology. Beyond their direct cytotoxic effects as ACPs, AMPs actively shape the immune landscape of the TME (70). Literature indicates that certain AMPs, such as human β-defensins and cathelicidin LL-37, function as chemo-attractants for dendritic cells, T cells, and monocytes, thereby enhancing immune cell infiltration and antigen presentation at the tumor site (71, 72). AMPs can also promote dendritic cell maturation and upregulation of co-stimulatory molecules, leading to more robust T-cell activation (72). Furthermore, some AMPs polarize tumor-associated macrophages from a pro-tumorigenic macrophage M2 phenotype toward an anti-tumor M1 state, fostering an inflammatory milieu conducive to tumor clearance (73).

AMPs modulate cytokine networks by inducing pro-inflammatory signals, e.g., interleukin-12 (IL-12), TNF-α, and interferon-gamma (IFN-γ) while suppressing immunosuppressive factors like IL-10 and transforming growth factor-beta (TGF-β) (74). Certain AMPs have been shown to enhance the cytotoxic function of NK and CD8^+^ T cells, protecting them from exhaustion within the immunosuppressive niche (75). These immunomodulatory properties are complemented by the adjuvant-like capacity of AMPs in vaccine formulations, where they stimulate durable immune memory against tumor antigens (76). Collectively, these functions position AMPs as promising agents not only for direct tumor cytotoxicity, but also for reprogramming the immune microenvironment to support sustained anti-tumor immunity—a dual mechanism that aligns closely with the objectives of modern CI, and provides a compelling rationale for their integration into combinational therapeutic strategies (77, 78).

## 5 CI: integration of AMPs

CI represents a paradigm shift in oncology, leveraging the body’s immune system to recognize, attack, and remember malignant cells. Unlike conventional therapies that directly target tumors, CI aims to enhance or restore intrinsic immune competence. Current modalities include immune checkpoint inhibitors, e.g., anti-programmed cell death protein/ programmed death-ligand 1 (anti-PD-1/PD-L1), anti-cytotoxic T-lymphocyte-associated protein 4 (anti-CTLA-4), chimeric antigen receptor (CAR) T-cell therapy, cancer vaccines, and cytokine therapies. Despite transformative successes, challenges persist, including variable patient response rates, immune-related adverse events, and the immunosuppressive TME (70).

Specific biomarkers, such as tumor antigen expression levels, immune cell infiltration profiles, and cytokine signatures, may serve as indicators of patient responses to AMP therapies. Identifying these biomarkers could facilitate personalized treatment approaches and enhance the therapeutic efficacy of AMPs.

The delivery route of AMPs significantly influences their therapeutic effectiveness and safety profiles. Intra-tumoral delivery can enhance local concentrations of peptides while minimizing systemic exposure, reducing potential side effects. However, systemic administration may be necessary for targeting metastatic disease, albeit with challenges related to peptide stability and bioavailability. Understanding the optimal delivery strategy is crucial for translating AMP therapies into clinical practice.

### 5.1 AMPs as direct ICD inducers

A foundational mechanism by which AMPs contribute to CI is through the induction of ICD. Unlike apoptotic or necrotic death, ICD is characterized by the release of damage-associated molecular patterns (DAMPs), such as calreticulin exposure on the cell surface, ATP secretion, and high mobility group box 1 (HMGB1) release. These signals act as potent adjuvants, recruiting dendritic cells and priming tumor-specific T-cell responses (79). Certain ACPs, such as LTX-315, have been specifically shown to induce ICD in melanoma and breast cancer models, leading to systemic antitumor immunity and abscopal effects on distant, untreated metastases (80). This positions AMPs not merely as local cytotoxins but as *in situ* cancer vaccines that can convert an immunologically “cold” tumor into a “hot,” T-cell-inflamed one.

### 5.2 AMPs as modulators of the TME

TME is often a major barrier to effective CI, characterized by immunosuppressive cells (e.g., regulatory T cells, M2 macrophages, myeloid-derived suppressor cells), inhibitory cytokines, and hypoxia. AMPs can directly remodel this hostile landscape. For instance, bovine neutrophil peptide indolicidin has been shown to repolarize M2 macrophages to a tumoricidal M1 phenotype. Similarly, human cathelicidin LL-37 can inhibit the recruitment and function of myeloid-derived suppressor cells (74). By breaking down these immunosuppressive networks, AMPs can enhance the infiltration and activity of adoptively transferred or endogenous cytotoxic T lymphocytes, thereby potentiating the efficacy of checkpoint blockade and CAR-T therapies (75).

### 5.3 AMPs as vaccine adjuvants and delivery vehicles

The development of therapeutic cancer vaccines relies on effective antigen presentation and the generation of durable T-cell memory. AMPs possess inherent adjuvant properties, activating dendritic cells via Toll-like receptors (e.g., TLR4) and other pattern recognition receptors (81). For example, the incorporation of defensins or synthetic AMP mimics into peptide or nucleic acid vaccine formulations has been shown to significantly enhance antigen-specific CD8^+^ T-cell responses and tumor protection in preclinical models (82). Beyond adjuvanticity, AMPs can function as self-assembling nanocarriers or conjugation partners for tumor antigens, improving their stability, cellular uptake, and cross-presentation, a key requirement for eliciting potent cytotoxic T-lymphocyte responses.

### 5.4 Combination strategies with established immunotherapies

Synergistic combinations of AMPs with existing CI agents are a promising frontier. Preclinical studies demonstrate that co-administration of AMPs with immune checkpoint inhibitors can overcome primary resistance. The mechanism involves AMP-mediated activation of dendritic cells and reversal of T-cell exhaustion, thereby creating a more permissive environment for checkpoint blockade (70, 83). Furthermore, AMPs can be engineered to selectively target tumor cells while sparing healthy tissues, reducing the systemic toxicity often associated with combination immunotherapies. For instance, tumor-homing peptides fused to AMP domains are being explored to concentrate immunomodulatory effects within the TME.

### 5.5 Challenges and translational considerations

Despite the compelling rationale for integrating AMPs into CI, translating these promising preclinical findings into clinical reality faces several hurdles that must be critically examined. Acknowledging these limitations provides a balanced perspective and frames the research priorities for the field.

#### 5.5.1 Biomarkers of response

A major barrier to the clinical success of AMP-based immunotherapies is the absence of validated biomarkers to guide patient selection and treatment monitoring. As with checkpoint inhibitors, where PD-L1 expression and tumor mutational burden serve as imperfect but useful predictive markers, AMP-based approaches will require analogous biomarkers to identify patients most likely to benefit. Candidate biomarkers under investigation include tumor-intrinsic factors such as the density of negatively charged membrane components (phosphatidylserine, O-glycosylated mucins) that determine AMP binding and lytic activity. Additionally, the baseline immune landscape may influence response, including tumor-infiltrating lymphocyte density, M1/M2 macrophage ratio, and expression of immune checkpoint molecules that could predict combinatorial benefit. Pharmacodynamic markers of AMP activity, such as evidence of ICD (circulating DAMPs including HMGB1, ATP, and calreticulin exposure) or changes in peripheral immune cell subsets, could demonstrate target engagement in early-phase trials. The prospective validation of such biomarkers will be essential for rational patient stratification and for demonstrating proof-of-mechanism in clinical studies.

#### 5.5.2 Delivery routes: intratumoral versus systemic administration

The route of AMP administration profoundly influences therapeutic index, pharmacokinetics, and immunological outcomes, yet optimal delivery strategies remain incompletely defined. Intratumoral delivery, involving direct injection into accessible tumor lesions, offers several advantages for immunomodulatory AMPs. This approach achieves high local concentrations while minimizing systemic exposure and off-target toxicity. Intratumoral administration of lytic peptides like LTX-315 has demonstrated the ability to induce ICD, release tumor antigens, and convert immunologically “cold” tumors into “hot” tumors responsive to checkpoint inhibitors (63). This strategy leverages the tumor itself as an *in situ* vaccine, potentially triggering broader immune responses against metastatic lesions through the abscopal effect. However, intratumoral delivery is limited to accessible tumors and may not adequately address widespread metastatic disease, raising questions about its applicability in advanced cancer settings.

In contrast, systemic delivery via intravenous or subcutaneous administration enables treatment of disseminated disease but faces significant hurdles. Rapid proteolytic degradation, renal clearance, and potential on-target off-tumor toxicity have historically limited the systemic use of peptide therapeutics. Strategies to overcome these limitations include PEGylation, nanoparticle encapsulation, D-amino acid substitution, cyclization, and conjugation to tumor-targeting moieties (20–24, 84, 85). Systemic delivery may be preferable for AMPs that function primarily through systemic immune modulation, such as cytokine induction or mobilization of peripheral immune effectors, rather than direct tumor lysis. The optimal delivery route ultimately depends on the AMP’s mechanism of action, therapeutic window, and disease context, with early-phase clinical trials exploring both approaches.

#### 5.5.3 Pharmacokinetic and toxicity hurdles

Beyond delivery route considerations, AMP-based immunotherapies face intrinsic pharmacological limitations. Susceptibility to proteolytic degradation in serum and rapid renal clearance necessitate frequent dosing or complex formulation strategies that may complicate clinical development and patient compliance. Furthermore, despite their selective toxicity toward cancer cells, some AMPs exhibit hemolytic activity or off-target effects on healthy tissues with negatively charged membranes, including liver and kidney, at therapeutic doses. These toxicity concerns have contributed to the attrition of several peptide candidates in clinical development.

#### 5.5.4 Clinical development status and lessons learned

Ongoing early-phase clinical trials are evaluating AMPs such as LTX-315 as intratumoral immunotherapies, with preliminary data indicating favorable safety and evidence of immune activation (63). However, it is instructive to examine why some peptide-based immunotherapies have advanced while others have failed. Successfully translated candidates typically possess optimized pharmacokinetic properties, well-defined mechanisms of action, and clear patient populations identified through biomarker strategies. Conversely, candidates that have faced challenges often encountered issues with bioavailability, narrow therapeutic windows, or failure to demonstrate superiority over evolving standards of care in phase III trials. For example, the development history of peptide vaccines like Nelipepimut-S illustrates how promising phase II results may not translate to phase III success without careful attention to patient selection and trial design (48) (see text footnote 24, 25). These lessons underscore that beyond potent mechanisms, successful clinical translation requires optimized formulation, appropriate delivery strategies, robust pharmacokinetic properties, and biomarker-driven patient stratification.

Addressing these challenges through rational drug design, innovative delivery systems, and rigorous clinical validation will be essential for realizing the therapeutic potential of AMPs as integral components of the immunotherapeutic arsenal.

## 6 Application of artificial intelligence algorithms for peptide design

Artificial intelligence (AI) algorithms (Table 3) integration has proven to be cost and time effective in computer-aided peptide drug designing, overcoming the challenges arising in traditional lab methods and screening (86). Among the effective tools for targeting and analyzing ACPs in terms of their structural details and characteristics are Random Forest (RF) and Support Vector Machine (SVM). The SVM-based AntiCP tool can predict the potential of a peptide to act as an ACP. The peptide’s anticancer potency can also be improved through providing a single point mutation for the input sequence, through utilizing features such as Amino Acid Composition (AAC) and Dipeptide Composition (DPC) (87). AntiCP2.0, a modified version of AntiCP which is Deep Learning (DL) based, is characterized by better prediction (88). Another DL-based tool, ACPred combines physical characteristics with several sequence-derived features, using convolutional neural networks (CNNs) for ACP prediction (89).

**Table 3:** Some ACP algorithms with their corresponding tools and features.

Algorithm	Tool	Accuracy	Feature	References
SVM	ACPP	96%	Compositional, centroidal, and distributional measures of amino acid residues	(108)
	iACP	92.67%	One gap dipeptide composition	(109)
	AntiCP	91.44%	Amino acid composition	(87)
			Dipeptide composition	
			Binary profile	
			Terminal profiling	
	ACPred-FL	91.4%	Composition–transition–distribution	(110)
			Amino acid composition	
			Adaptive skip dipeptide composition	
			G-gap dipeptide composition	
			Overlapping property	
			Binary profile	
			Twenty-one-bit	
RF	ACPred-fuse	89%	Amino acid composition	(111)
			G-amino acid composition	
			Dipeptide composition	
			G-dipeptide composition	
			G gap	
			Tripeptide composition	
			G-tripeptide composition	
			Composition–transition–distribution	
			K-spaced amino acid composition	
SVM/RF	ACPred	95.61%	Amino acid composition	(89)
			Pseudo amino acid composition	
			Dipeptide composition	
			Physiochemical properties	
			Amphiphilic pseudo amino acid composition	

Molecular docking can dive through ACP interactions with the target binding proteins with respect to binding affinity, specificity and stability, predicting the most favorable position of the ligand attached to the receptor’s active site, and its binding strength. This leads to an accurate selection of the best peptide candidate for synthesis and testing, in addition to virtual screening of large libraries in short time spans (90). Among the highest-ranking molecular docking tools are Glide, Auto Dock Vina, and Auto Dock GOLD (91, 92).

Generative AI plays a transforming role in low-cost accelerated drug discovery, through designing peptide sequences with therapeutic properties and minimizing experimental workload. This is achieved through learning the underlying patterns of protein sequences and structures from huge sets of biological data. The leveraging architectures employed include CNN, variational autoencoders (VAE), generative adversarial networks (GAN), diffusion models, protein language models (PLM) and reinforcement learning (RL). VAE and CNN generate diverse peptide candidates, GAN incorporates structural context into design, RL provides goal-directed optimization of peptides toward desired properties, whereas PLM and diffusion models create highly specific peptide therapeutics through learning the fundamental rules of protein structure and function (93).

Despite the achieved advancements in this field, a challenge still resides in bridging the gap between in silico predictions and successful *in vivo* applications, manifested in poor prediction of vital parameters crucial for the peptides activities, such as stability towards proteolytic degradation, serum binding affinity, clearance rates, and efficacy of delivery to the target site. In that context, liposomes and nano-carriers are being introduced to overcome such challenges (94).

## 7 Conclusion

AMPs have emerged as a versatile and promising class of agents in the fight against cancer, functioning not only as direct cytotoxic ACPs, but also as potent immunomodulators. Their unique ability to selectively target and disrupt cancer cell membranes, owing to the heightened negative charge of malignant cells, sets them apart from traditional therapies by minimizing off-target toxicity and overcoming drug resistance. Through diverse mechanisms such as membrane disruption, intracellular interference, and induction of ICD, AMPs offer a multifaceted approach to tumor eradication that complements and enhances conventional treatment modalities.

Beyond their direct antitumor effects, AMPs play a critical role in reshaping the TME and augmenting the body’s immune response. By attracting immune cells, promoting dendritic cell maturation, polarizing macrophages toward an anti-tumor phenotype, and modulating cytokine networks, AMPs help convert immunologically “cold” tumors into “hot,” T-cell-inflamed environments. This immunomodulatory capacity aligns closely with the goals of modern CI, providing a strategic means to overcome the immunosuppressive barriers that often limit the efficacy of existing immunotherapies.

The integration of AMPs into CI frameworks, such as *in situ* vaccines, vaccine adjuvants, or combination partners with checkpoint inhibitors and CAR-T therapies, has demonstrated significant synergistic potential in preclinical models. However, it is important to critically evaluate the current evidence base. Many studies demonstrating AMP efficacy remain at the preclinical stage, with limited validation in robust *in vivo* models that accurately recapitulate human disease. Much of the mechanistic understanding, particularly regarding immune modulation, derives from *in vitro* experiments that may not fully reflect the complexity of the TME in patients. Furthermore, the heterogeneity of cancer cells, both between patients and within individual tumors, raises questions about the universality of AMP selectivity, as not all malignant cells display the membrane characteristics required for optimal AMP binding and activity.

Several key areas of uncertainty persist and warrant further investigation. First, the precise relationship between AMP structure, membrane composition, and selective cytotoxicity remains incompletely understood, limiting rational design efforts. Second, while AMPs show promise as immunomodulators, the durability and specificity of the immune responses they generate have not been systematically characterized. Third, optimal dosing, scheduling, and sequencing with conventional therapies and other immunotherapies remain largely undefined, with most combination studies to date relying on empirical rather than mechanistic rationale. Fourth, the extent to which preclinical findings will translate to human patients, given differences in pharmacokinetics, immune system complexity, and tumor biology represents a fundamental uncertainty that can only be resolved through rigorous clinical investigation.

Translational challenges such as peptide stability, systemic toxicity, and optimized delivery must be addressed through continued innovation in peptide engineering, nanoformulation, and targeted delivery systems. Moreover, the field would benefit from standardized assays to compare AMP candidates, validated biomarkers to guide patient selection, and well-designed clinical trials that test mechanistically driven hypotheses rather than empirical combinations.

In summary, AMPs possess genuine potential as dual-function agents that bridge direct tumor killing with immune activation. However, realizing this potential will require moving beyond descriptive preclinical studies toward hypothesis-driven research that addresses fundamental questions about mechanism, specificity, and *in vivo* behavior. The next decade will be critical in determining whether the promise of AMPs as cancer therapeutics can be translated into meaningful clinical benefit for patients with resistant or advanced malignancies.

## 8 Future perspectives and potentials

Looking forward, the field of AMP-based cancer therapy holds substantial promise but requires focused efforts to overcome existing barriers and fully realize its clinical potential. Future research should prioritize the rational design of synthetic and hybrid AMPs with enhanced stability, reduced hemolytic activity, and improved tumor specificity through modifications such as D-amino acid substitution, cyclization, and conjugation to targeting moieties. Additionally, advanced delivery systems, including nanoparticles, hydrogels, and stimulus-responsive carriers, will be crucial for prolonging circulation time, enabling controlled release, and minimizing systemic exposure. The development of non-peptidic AMP mimetics may further circumvent limitations related to proteolytic degradation and immunogenicity.

From an immunological perspective, deeper mechanistic studies are needed to elucidate how AMPs interact with specific immune cell subsets and signalling pathways within the TME. Combining AMPs with emerging modalities such as oncolytic viruses, bispecific antibodies, or epigenetic modulators could unlock novel synergistic effects and broaden their applicability across cancer types. Moreover, biomarker-driven clinical trials will be essential to identify patient populations most likely to benefit from AMP-based therapies and to tailor combinations accordingly.

In the longer term, AMPs may also contribute to preventive and neoadjuvant strategies, potentially serving as vaccine components or immune primers in high-risk individuals. As our understanding of the tumor–immune interface grows, AMPs are poised to become integral tools in the development of multimodal, precision immunotherapy regimens. With sustained interdisciplinary collaboration and translational investment, AMP-based approaches could significantly advance oncology toward more effective, durable, and safer treatments for cancer patients worldwide.

## Footnotes

1 https://go.drugbank.com/drugs/DB06719 [accessed October 16, 2025].

2 https://pubchem.ncbi.nlm.nih.gov/compound/50225#section=Mechanism-of-Action [accessed October 16, 2025]

3 https://go.drugbank.com/drugs/DB00970 [accessed October 16, 2025].

4 https://pubchem.ncbi.nlm.nih.gov/#query=Dactinomycin [accessed October 16, 2025].

5 https://go.drugbank.com/drugs/DB04977 [accessed October 16, 2025].

6 https://pubchem.ncbi.nlm.nih.gov/compound/9812534 [accessed October 16, 2025].

7 https://go.drugbank.com/drugs/DB15283 [accessed October 16, 2025].

8 https://pubchem.ncbi.nlm.nih.gov/#query=Triptorelin [accessed October 16, 2025].

9 https://go.drugbank.com/drugs/DB06825 [accessed October 16, 2025].

10 https://pubchem.ncbi.nlm.nih.gov/compound/9960285#section=Biologic-Description [accessed October 16, 2025].

11 https://go.drugbank.com/drugs/DB05491 [accessed October 16, 2025].

12 https://pubchem.ncbi.nlm.nih.gov/#query=Canfosfamide [accessed October 25, 2025].

13 https://go.drugbank.com/drugs/DB04972 [accessed October 25, 2025].

14 https://pubchem.ncbi.nlm.nih.gov/#query=CTCE-0214 [accessed October 25, 2025].

15 https://go.drugbank.com/drugs/DB05934 [accessed October 25, 2025].

16 https://pubchem.ncbi.nlm.nih.gov/#query=darinaparsin [accessed October 25, 2025].

17 https://go.drugbank.com/drugs/DB06179 [accessed October 25, 2025].

18 https://pubchem.ncbi.nlm.nih.gov/#query=IRL-1620 [accessed November 1, 2025].

19 https://go.drugbank.com/drugs/DB06138 [accessed November 1, 2025].

20 https://pubchem.ncbi.nlm.nih.gov/#query=Iseganan [accessed November 2, 2025].

21 https://go.drugbank.com/drugs/DB16010 [accessed November 2, 2025].

22 https://pubchem.ncbi.nlm.nih.gov/#query=Labradimil [accessed November 2, 2025].

23 https://go.drugbank.com/drugs/DB06549 [accessed November 2, 2025].

24 https://pubchem.ncbi.nlm.nih.gov/#query=Nelipepimut-S [accessed November 2, 2025].

25 https://go.drugbank.com/drugs/DB06226 [accessed November 2, 2025].

26 https://pubchem.ncbi.nlm.nih.gov/#query=PM02734 [accessed November 5, 2025].

27 https://go.drugbank.com/drugs/DB05158 [accessed November 2, 2025].

28 https://go.drugbank.com/drugs/DB05210 [accessed November 5, 2025].

29 https://www.medchemexpress.com/sf1126.html?srsltid=AfmBOoqAcnonmex0lagIUJ_zziutEP2yuf4U--TuGOaoFo6swrhDKzy3 [accessed November 5, 2025].

30 https://pubchem.ncbi.nlm.nih.gov/#query=Teverelix [accessed November 12, 2025].

31 https://www.medchemexpress.com/search.html?q=Teverelix&ft=&fa=&fp=&fsp=&ftag=&fsc=&fcc=&fol=&type=inhibitors-and-agonists [accessed November 12, 2025].

32 https://pubchem.ncbi.nlm.nih.gov/#query=Tigapotide [accessed November 12, 2025].

33 https://go.drugbank.com/drugs/DB04985 [accessed November 12, 2025].

34 https://pubchem.ncbi.nlm.nih.gov/#query=Balixafortide [accessed November 12, 2025].

35 https://go.drugbank.com/drugs/DB15370 [accessed November 12, 2025]

36 https://pubchem.ncbi.nlm.nih.gov/#query=Bleomycin%20A6 [accessed November 12, 2025].

37 https://go.drugbank.com/drugs/DB12992 [accessed November 12, 2025].

38 https://pubchem.ncbi.nlm.nih.gov/#query=Bombesin [accessed November 27, 2025].

39 https://go.drugbank.com/drugs/DB11724 [accessed November 27, 2025].

40 https://pubchem.ncbi.nlm.nih.gov/#query=Dolastatin%2010 [accessed November 28, 2025].

41 https://go.drugbank.com/drugs/DB12730 [accessed November 28, 2025].

42 https://pubchem.ncbi.nlm.nih.gov/#query=Soblidotin [accessed November 28, 2025].

43 https://go.drugbank.com/drugs/DB12677 [accessed November 28, 2025].

44 https://pubchem.ncbi.nlm.nih.gov/#query=LTX-315 [accessed November 28, 2025].

45 https://go.drugbank.com/drugs/DB12748 [accessed November 28, 2025].

46 https://pubchem.ncbi.nlm.nih.gov/#query=Ozarelix [accessed November 28, 2025].

47 https://go.drugbank.com/drugs/DB12581 [accessed November 28, 2025].

48 https://pubchem.ncbi.nlm.nih.gov/#query=TAK-448 [accessed November 29, 2025].

49 https://go.drugbank.com/drugs/DB11975#BE0014712 [accessed November 29, 2025].

50 https://pubchem.ncbi.nlm.nih.gov/#query=Valspo [accessed November 29, 2025].

51 https://go.drugbank.com/drugs/DB11869 [accessed November 29, 2025].

52 https://pubchem.ncbi.nlm.nih.gov/#query=VEGFR2%E2%80%93169 [accessed November 29, 2025].

53 https://go.drugbank.com/drugs/DB16152 [accessed November 29, 2025].

54 https://pubchem.ncbi.nlm.nih.gov/#query=Zoptarelin%20doxorubicin [accessed November 29, 2025].

55 https://go.drugbank.com/drugs/DB12755 [accessed November 29, 2025].

## Glossary

AAC: Amino Acid Composition
ACP: anticancer peptide
AI: Artificial intelligence
AMP: antimicrobial peptide
ATP: adenosine triphosphate
CAR: chimeric antigen receptor
CD8⁺ T: cytotoxic T lymphocytes
CI: cancer immunotherapy
CNNs: convolutional neural networks
CTLA-4: cytotoxic T-lymphocyte-associated protein 4
CXCR4: chemokine CXC receptor type 4
DAMPs: damage-associated molecular patterns
DL: deep learning
DNA: deoxyribonucleic acid
DPC: Dipeptide composition
DR: death receptor
eEF1A2: elongation factor 1A2
EMA: European Medicines Agency
FDA: Food and Drug Administration
GAN: generative adversarial networks
GnRH: gonadotrophin-releasing hormone
GnRHR: gonadotrophin-releasing hormone receptor
Gp100: glycoprotein100
GST P1-1: glutathione S-transferase P1-1
HER2/neu: human epidermal growth factor receptor 2/neuroblastoma
HLA-A: class I histocompatibility antigen, A alpha chain
HMGB1: high mobility group box 1
ICD: immunogenic cell death
IFN-γ: interferon-gamma
IL-10: interleukin-10
IL-12: interleukin-12
ImmTACs: immune-mobilizing monoclonal T cell receptors against cancer
LHRH: luteinizing hormone-releasing hormone
NB4: human acute promyelocytic leukemia cell line
NK: natural killer cells
NKT: natural killer T cells
PD-1: programmed cell death protein
PD-L1: programmed death-ligand 1
PG-1: protegrin-1
PLM: protein language models
RF: random forest
RGD: arginylglycylaspartic acid
RNA: ribonucleic acid
SDF-1α: stromal cell-derived factor
SVM: support vector machine
TGF-β: transforming growth factor-beta
TLR: Toll-like receptors
TME: tumor microenvironment
TNF-α: tumor necrosis factor-alpha
TRAIL: tumor necrosis factor-related apoptosis-inducing ligand
VAE: variational autoencoders
VEGFR: vascular endothelial growth factor receptor
